# Quantum gas magnifier for sub-lattice-resolved imaging of 3D quantum systems

**DOI:** 10.1038/s41586-021-04011-2

**Published:** 2021-11-24

**Authors:** Luca Asteria, Henrik P. Zahn, Marcel N. Kosch, Klaus Sengstock, Christof Weitenberg

**Affiliations:** 1 Institut für Laserphysik, Universität Hamburg, Hamburg, Germany; 2 The Hamburg Centre for Ultrafast Imaging, Hamburg, Germany; 3 Zentrum für Optische Quantentechnologien, Universität Hamburg, Hamburg, Germany

**Keywords:** Bose-Einstein condensates, Quantum simulation

## Abstract

Imaging is central to gaining microscopic insight into physical systems, and new microscopy methods have always led to the discovery of new phenomena and a deeper understanding of them. Ultracold atoms in optical lattices provide a quantum simulation platform, featuring a variety of advanced detection tools including direct optical imaging while pinning the atoms in the lattice^1,2^. However, this approach suffers from the diffraction limit, high optical density and small depth of focus, limiting it to two-dimensional (2D) systems. Here we introduce an imaging approach where matter wave optics magnifies the density distribution before optical imaging, allowing 2D sub-lattice-spacing resolution in three-dimensional (3D) systems. By combining the site-resolved imaging with magnetic resonance techniques for local addressing of individual lattice sites, we demonstrate full accessibility to 2D local information and manipulation in 3D systems. We employ the high-resolution images for precision thermodynamics of Bose–Einstein condensates in optical lattices as well as studies of thermalization dynamics driven by thermal hopping. The sub-lattice resolution is demonstrated via quench dynamics within the lattice sites. The method opens the path for spatially resolved studies of new quantum many-body regimes, including exotic lattice geometries or sub-wavelength lattices^3–6^, and paves the way for single-atom-resolved imaging of atomic species, where efficient laser cooling or deep optical traps are not available, but which substantially enrich the toolbox of quantum simulation of many-body systems.

## Abstract (web summary)

Existing techniques for high-resolution imaging of trapped quantum gases are limited to two-dimensional systems, but the approach described here works in three dimensions by magnifying the quantum gas with matter wave optics.

## Main

Experimentally driven understanding of quantum mechanical phenomena depends crucially on the possibility of observing them at the microscopic level. The quantum nature of matter shows itself on small scales, which has triggered tremendous efforts to develop advanced methods with increasing resolution to image the quantum system itself. Here, we introduce the alternative approach based on the idea to first magnify the quantum system itself to more accessible scales, which can then be easily imaged. We demonstrate this approach in a quantum simulator composed of quantum gases in the form of ultracold atoms in optical lattices and realize imaging of 3D systems with 2D sub-lattice resolution.

Direct optically resolved imaging of ultracold atoms in optical lattices, known as quantum gas microscopy^1,2^, requires very high numerical apertures and is so far restricted to 2D systems due to the fundamental limitation of the depth of focus and to unit lattice site occupation due to light-assisted collisions. The depth of focus can be overcome by using an electron microscope^7^ or an ion microscope^8^, but at the cost of a reduced detection efficiency and a large technological complexity. Recent experiments have reached sub-lattice resolution via super-resolution microscopy using nonlinear atom–light interactions^9,10^, but relying on scanning techniques. Our quantum gas magnifier does not suffer from these limitations and extends 2D sub-lattice-site-resolved imaging to new 3D regimes such as bosons or fermions in 3D optical lattices or sub-wavelength lattices with drastically enhanced energy scales^3–6^. The technique yields full single-shot images, which gives direct access to density correlations and, for example, spontaneous pattern formation such as density waves. Furthermore, the concept can be applied and adapted to very different physical systems such as exotic atomic species or mixtures.

**Fig. 1:**
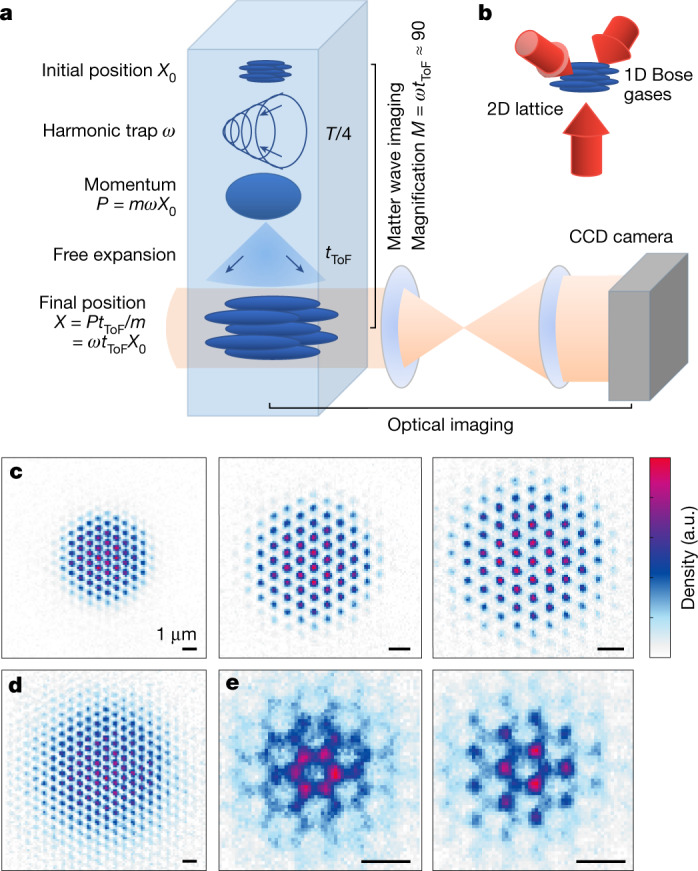
Working principle of the quantum gas magnifier using matter wave optics. **a**, The density distribution of ultracold atoms in an optical lattice is magnified by matter wave optics composed of a pulsed dynamics in a harmonic trap and a free expansion. Subsequently, it can be imaged with optical absorption imaging of moderate resolution and without restrictions from optical density or depth of focus. **b**, Sketch of the 2D hexagonal optical lattice. **c**, Images of ultracold bosonic atoms in a 2D triangular lattice for constant system size given by the confinement *ω*_system_/2π = 225 Hz, but varying magnification of *M* = 43(1), 65(1), 80(1) (from left) tuned via tighter magnetic confinements *ω*_pulse_ during the matter wave optics. **d**, Image of a larger system with confinement *ω*_system_/2π = 89 Hz imaged with magnification *M* = 43(1). **e**, Images of a honeycomb lattice and a boron nitride lattice with a sublattice offset of 4.6 kHz with a magnification of *M* = 89(1). The scale bars have a length of 1 µm. The atom number is in between 48,000 and 59,000 for the six images.

Our quantum gas magnifier uses matter wave optics in the time domain to magnify the atomic density distribution before the standard optical absorption imaging^11,12^. To this end, a harmonic potential of trapping frequency *ω*_pulse_ = 2π/*T* is applied for a time *T*/4, mapping the spatial distribution to the momentum distribution^13–16^. This is initialized in our case by switching off the lattice, which additionally helps in limiting interaction-driven aberrations due to the fast decrease in local density (see Supplementary Information). This matter wave lens is followed by free time-of-flight expansion (ToF) of duration *t*_ToF_. This combination reproduces the initial spatial distribution with a magnification *M* ≈ *ω*_pulse_*t*_ToF_ (Fig. 1a). Note that more complex pulsed lenses and other time-domain optical elements can be used in this scheme as well. An advantage of combining a *T*/4 pulse with time of flight is that the aberrations introduced by the finite ToF can be perfectly compensated by choosing the evolution time in the harmonic trap slightly above *T*/4 (see Supplementary Information).

Figure 1c–e demonstrates the power of this method with the first single-shot site-resolved images of a 3D quantum gases in 2D optical lattices including images of lattices with two-atomic basis. In the following, after describing the concept more closely, we additionally demonstrate high-resolution thermometry across the thermal-to-Bose–Einstein condensate (BEC) phase transition for a 3D quantum gas in a triangular optical lattice as well as full local addressability and precision measurements of thermally activated dynamics in a lattice system. Finally, we also demonstrate sub-wavelength resolution to study local dynamics. The flexibility and adaptability of our concept now allow for very precise locally resolved and locally controlled measurements of higher-dimensional quantum gas systems.

The experiments presented here use ^87^Rb BECs evaporatively cooled in a magnetic trap. The potential of the magnetic trap is in-plane radially symmetric with a trapping frequency which is ramped within 100 ms to *ω*_system_ = 2π × [89−658]  Hz. We ramp up triangular or honeycomb optical lattices formed by the interference of lattice beams of wavelength *λ* = 1,064 nm leading to a lattice constant of *a*_lat_ = 2*λ*/3 = 709 nm, which sets the energy scale *E*_rec_ = *h*^2^/(2*mλ*^2^) for the lattice depth, where *h* is Planck’s constant and *m* the atomic mass. The harmonic transverse confinement has a trapping frequency *ω*_*z*_ of typically 2π × 29 Hz, resulting in a Josephson junction array of BECs in the tubes of the 2D lattice. The trap frequency is then ramped to *ω*_pulse_ for the magnification protocol after freezing the density distribution in a deep optical lattice (see Supplementary Information). The magnetic trap is suitable for the *T*/4 evolution because of its smoothness, radial symmetry and strong confinement: for typical parameters of *t*_ToF_ ≈ 20 ms and *ω*_pulse_/(2π) up to ~700 Hz we measure large magnifications of up to *M* = 93(1), allowing resolution of the lattice spacing with conventional absorption imaging with magnification 2 on a charge-coupled device (CCD) camera (Fig. 1). The uncertainty in parentheses corresponds to the 68% statistical confidence interval.

The resolution of the quantum gas magnifier can be made very high because the harmonic trap has a large spatial extension corresponding to a large numerical aperture of the matter wave optics. In practice the resolution is mainly limited by the convolution with our optical imaging resolution (see Supplementary Information). The effect of interactions during the magnification protocol can be effectively suppressed by working with incoherent systems or by removing the coherence via freezing in a deep lattice (see Supplementary Information).

As a first benchmark experiment, we study the thermal-to-BEC phase transition in a lattice of tubes, allowing us to confirm the faithful imaging of lattice site occupations. Furthermore, we show how the high-resolution access to real-space profiles via the magnifier provides an excellent approach to optical lattice thermometry, which requires much greater numerical effort from the more common momentum space images^17–19^.

**Fig. 2:**
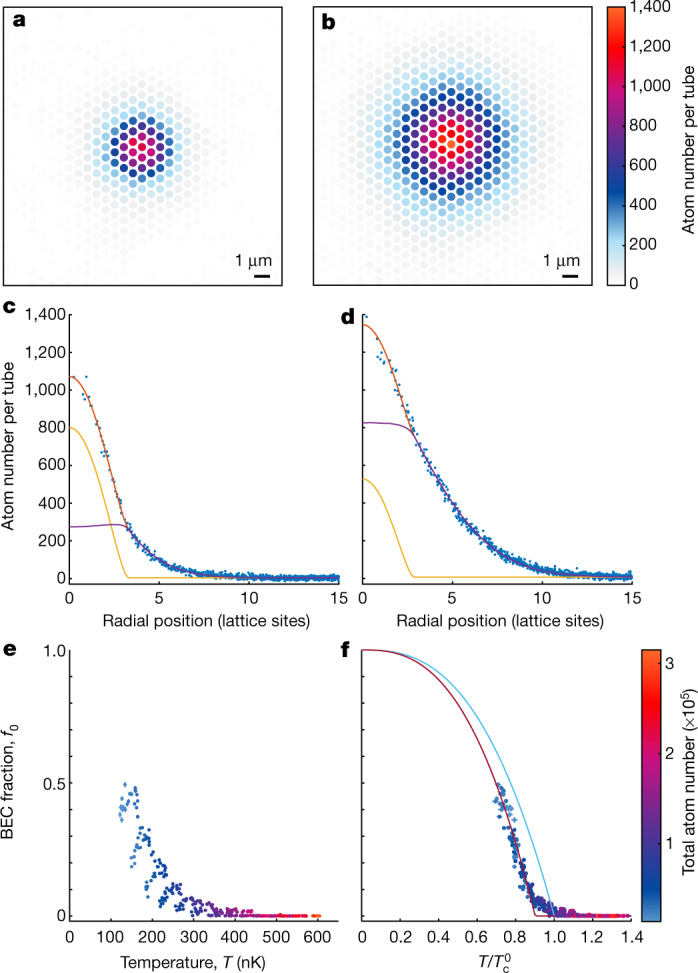
Thermal-to-BEC phase transition in optical lattices observed via high-resolution density profiles. **a**, **b**, Spatial density distributions of BECs in triangular optical lattices prepared at different temperatures and atom numbers of 171(1) nK and 37,000(400) atoms (**a**), and 310(1) nK and 106,000(600) atoms (**b**). The densities are shown as atom numbers per lattice site from integration over the Wigner–Seitz cells. **c**, **d**, Atom number per tube as a function of the radial position corresponding to **a** and **b**, respectively, with a bimodal fit (orange line) consisting of the condensed part (yellow line) and the thermal part (purple line). **e**, **f**, Condensate fraction (circles) obtained from the bimodal fits as a function of the temperature (**e**) and of the temperature in units of the scaling temperature ${T}_{{\rm{c}}}^{0}$ (**f**). Most error bars are smaller than the symbol size. The light-blue line in **f** shows the power law approximation of the non-interacting theory described in the main text. The purple line is a fit to the data with the same power law. The bandwidth of the lowest band is *k*_B_ × 5.4 nK and the gap between the first and second band is *k*_B_ × 290 nK. The colour encodes the total atom number of the clouds. All error bars correspond to the 68% confidence interval. Source data

To study the phase transition, we prepare the system at varying temperature and atom number by adjusting the end point of the evaporation ramp and a varying hold time before ramping up the lattice to the final depth with tunnelling energy *J* = *h* × 12 Hz. For the analysis, we start with the extraction of the on-site populations (Fig. 2a, b). The data can be described by a bimodal model consisting of a condensed part and a thermal part including the repulsion of the thermal atoms from the condensate in mean-field approximation (see Methods). The model is fitted to the 2D distribution and the excellent fit quality can be seen when plotting the data as a function of the radial position (Fig. 2c, d) confirming the exact measurement of the lattice site occupations.

The fit allows us to extract the temperature *T* from the thermal component and the condensate fraction *f*_0_ from the atom numbers in the two components with very high precision. Owing to the dependence of the critical temperature *T*_c_ on the total atom number, the condensate fraction as a function of temperature does not result in a single curve (Fig. 2e). To describe this dependence we set up an analytic non-interacting model predicting the critical temperature ${T}_{{\rm{c}}}^{0}$ to renormalize the experimental temperatures using ${T}_{{\rm{c}}}^{0}$ as a scaling temperature, resulting in a collapse of the data on a single curve (Fig. 2f). We observe a shift of the critical temperature towards lower values compared to the non-interacting model. To quantify this shift we approximate the non-interacting model by a power law in the density of states, resulting in a description *f*_0_ = 1 −( *T*/*T*_c_)^*α*^ with *α* = 2.69(1) characterizing the underlying density of states interpolating between a lattice regime and a continuum regime (see Methods).

Fitting this function to the data satisfying *f*_0_ > 0.1 results in ${T}_{{\rm{c}}}=0.901(4){T}_{{\rm{c}}}^{0}$, where the small statistical error reflects the excellent collapse on a single curve, thus showing the quality of the thermometry. Additionally, we estimate a systematic error of 1% stemming from an uncertainty of the atom number calibration of 3%. A shift of this order of magnitude is expected from interactions and finite size^20^, but a closed theoretical model for our regime where both trap and lattice are relevant does not exist. With the enhanced interactions in the optical lattice, the shift is larger than those experimentally observed for BECs in 3D harmonic traps for comparable atom numbers^21,22^. Interestingly, we observe a pronounced smoothing of the phase transition despite the rather large atom number, which might be due to the 2D–3D crossover geometry of an array of tubes. Our precision thermometry measurements thus provide a benchmark for future theoretical studies of phase transitions in such geometries.

In a second set of experiments, we employ magnetic resonance (MR) techniques to realize local addressing of individual lattice sites^23^ and thereby demonstrate the full functionality of quantum gas microscopes without the need for large optical access thus making it compatible with other experimental constraints. While site-resolved addressing was previously also realized optically^24,25^ and with an electron beam^26^, MR techniques are optimally suited for 3D systems by avoiding the depth of focus limitation of optical addressing beams and have, for example, been proposed for wavefunction engineering^27^.

**Fig. 3:**
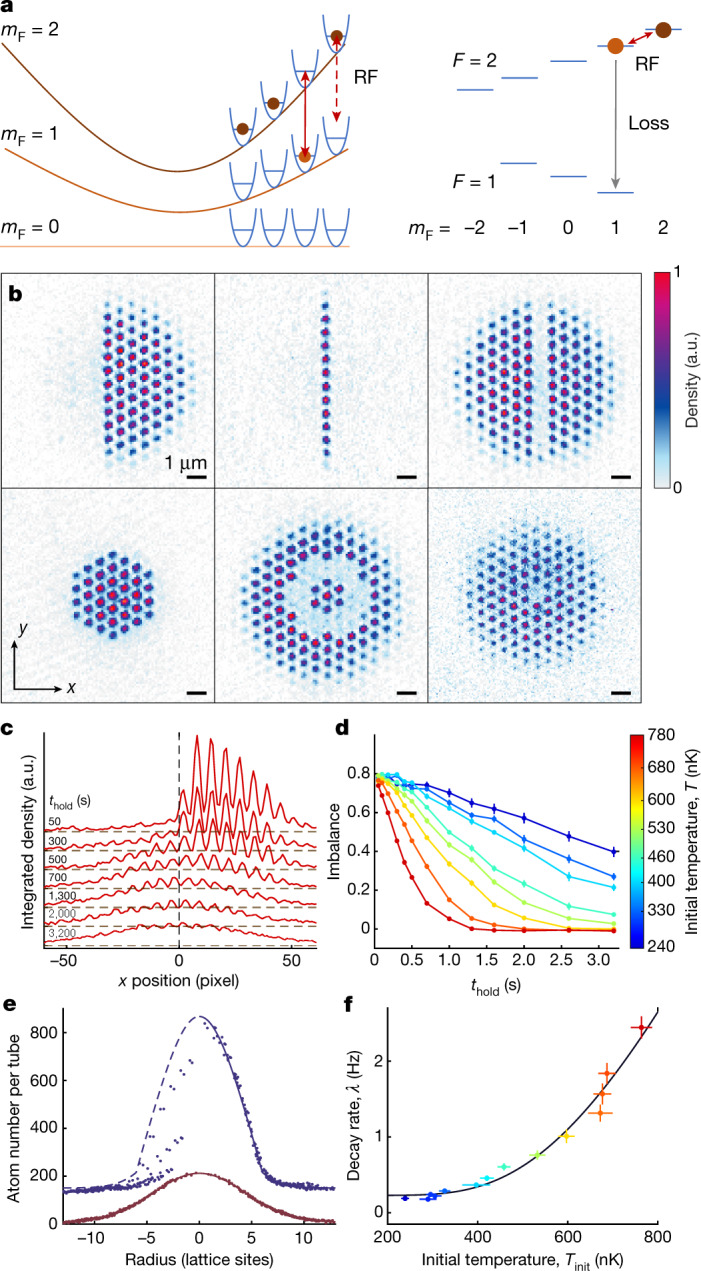
Local addressing and thermalization dynamics. **a**, Scheme of local addressing via RF transitions in a magnetic field gradient and a sketch of the hyperfine states of ^87^Rb with the utilized RF transition and the loss channel. **b**, Example images of prepared density distributions. In the upper row, the magnetic trap is shifted approximately two times the system diameter along the *x* direction before applying the RF sweeps. In the lower row, the magnetic trap is not shifted. The magnification is 60(1) for the first five images and 77(1) for the last image. **c**, Single-shot density profiles integrated along the *y* direction for different hold times after removing the left half of the cloud illustrating the thermalization dynamics. The different profiles are offset for clarity. The initial temperature is *T*_init_ = 0.76(2) μK. **d**, Time evolution of the imbalance for different initial temperatures. **e**, Density as a function of radial position after 50 ms (blue), shifted upwards for clarity, and 3.2 s (red) hold time with bimodal fits using only lattice site populations with positive *x* positions larger than the maximally populated line (blue) and all populations (red) respectively. The data are averaged over 27 images (including the top and bottom data from **c**). The fit yields temperatures of 0.68(5) μK and 1.25(4) μK and demonstrates the reached thermal equilibrium. **f**, Dependence of the decay time on the initial temperature modelled by an Arrhenius process for thermal hopping and an offset rate for quantum tunnelling (see text and Methods). All error bars correspond to the 68% confidence interval. Source data

In the experimental protocol, we freeze the atomic distribution in a deep lattice and shift the magnetic trap (*ω*_addressing_/2π = 543 Hz) by up to 20 µm, creating magnetic gradients between 23 and 50 kHz µm^−1^ at the atom’s position. The magnetic gradient spatially splits the radio frequency (RF) transition between the initial stretched *F* = 2, *m*_F_ = 2 state and the target *F* = 2, *m*_F_ = 1 state and we drive spin flips at positions controlled via RF sweeps (Fig. 3a). To empty the addressed lattice sites, we make use of the strongly spin-dependent loss rates driven by hyperfine-changing collisions^28^, which are suppressed for the stretched initial spin state but empty the addressed lattice sites during the sweep time of 100–400 ms. When choosing *F*-changing transitions instead, the removal of one state could be achieved via an optical push out. The magnifier approach can also be easily extended to spin dependent imaging (see Supplementary Information). By choosing the appropriate RF sweeps addressing equipotential surfaces of the magnetic trap, we create very well resolved patterns such as rings of varying radius or—when placing the atoms at the slope of the magnetic trap—single lines or half systems (Supplementary Information) (Fig. 3b).

Subsequently, we probe the thermalization dynamics after removal of atoms from one half of the system^29^ (Fig. 3c). We monitor the thermalization via the imbalance ${\mathcal I} =({N}_{{\rm{R}}}-{N}_{{\rm{L}}})/({N}_{{\rm{R}}}+{N}_{{\rm{L}}})$ defined as the relative difference of the atom numbers *N*_R_ in the right half and *N*_L_ in the left half of the trap. The imbalance ${\mathcal I}$ decays to zero (Fig. 3c, d) and we determine the thermalization rate from an exponential fit. We verify that the profiles with no imbalance are indeed in thermal equilibrium (Fig. 3e) by fitting a bimodal model consisting of an inverted parabola and a Gaussian.

The thermalization rate as a function of the initial temperature is almost constant up to temperatures of about 350 nK and then increases steeply with temperature (Fig. 3f). We model this by an Arrhenius law describing thermal hopping combined with an offset rate resulting from quantum tunnelling (see Methods). We obtain a potential barrier height of *V*_B_ = *k*_B_ × 2.4(6) µK, where *k*_B_ is the Boltzmann constant, in excellent agreement with the peak-to-peak lattice depth of *k*_B_ × 2.6 µK deduced from lattice depth calibration and an offset rate *Γ*_0_ = 0.23(8)  Hz related to the tunnelling energy *J* = *h* × 0.1 Hz of the lowest band. These experiments demonstrate that the quantum gas magnifier allows very precise spatially resolved studies of thermalization dynamics in optical lattices in new parameter regimes, which could be extended to strongly correlated regimes by adding a transverse lattice.

**Fig. 4:**
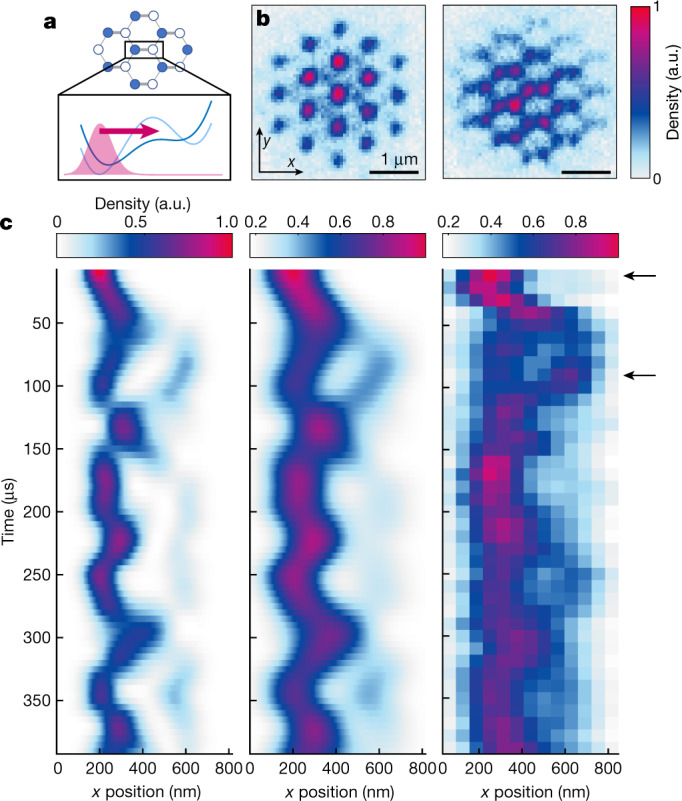
Nanoscale dynamics in a honeycomb optical lattice. **a**, The honeycomb lattice with energy offset between the A sites (closed circles) and B sites (open circles) can be tuned into a lattice of dimers with stronger tunnelling bonds along one direction (thicker grey lines). The inset shows cuts of the potential along a dimer before (light blue line) and after the quench (dark blue line) together with the initial density profile (red area). **b**, Experimental images for 10 µs, 90 µs after the quench. A lattice vector corresponds to 10.9 pixels with a magnification of *M* = 93(1). **c**, Time evolution of the density distributions within one dimer after the quench from the simulation (left) and from the experiment (right, cut of 1 pixel width). For a realistic comparison we have broadened the simulation results with a Gauss filter of 76 nm width and added an offset (see Methods) (middle). The arrows mark the evolution times shown in **b**. Source data

Finally, we demonstrate the capability to resolve density features well below the lattice spacing by observing nanoscale dynamics after a quench of the lattice geometry. We start in a deep honeycomb lattice with large sublattice offset (see Methods) leading to an initial population of the A sublattice only and control the geometry by varying the imbalance of the lattice beam intensities *I*_1_, *I*_2_ and *I*_3_. By abruptly reducing *I*_2_ = *I*_3_ to 0.5*I*_1_, we create a lattice of dimers with enhanced tunnel coupling within the dimer as well as a displacement of the lattice sites (Fig. 4a), thus exciting both a tunnelling oscillation between the A and B sites and an oscillation within the lattice sites.

The resulting dynamics of the atomic density within the dimer (averaged over all dimers with at least 50% of the signal in the most populated dimer) is shown in (Fig. 4b, c). We capture the dynamics by a non-interacting multi-band simulation including the finite switching time of the laser intensities of about 20 μs. The quantum gas magnifier on honeycomb optical lattices allows resolving the interplay oftunnelling dynamics between lattice sites with nanoscale dynamics within the lattice sites^9,10^ and opens a real-space approach to studying multi-orbital systems especially for extended 3D systems.

In conclusion, we have introduced a quantum gas magnifier based on matter wave optics and used it to image 3D quantum gases in triangular and honeycomb optical lattices with a resolution below the lattice spacing. Spatially resolved measurements give access to central scientific problems such as transport phenomena^29^, spontaneous domain formation^30^, or chiral edge and interface states in interacting topological matter^31^. We estimate that the method can be pushed to a single-atom sensitive regime using free-space fluorescence imaging after the matter wave magnification, when the magnified lattice spacing is larger than the diffusive expansion from photon scattering^32,33^ or using metastable helium and multi-channel plates^34^ (see Supplementary Information). This will allow for a direct study of correlations in strongly interacting systems of atomic species, for which laser cooling and very deep optical lattices as in conventional quantum gas microscopes are not available. The magnification approach also circumvents pairwise atom loss during imaging in quantum gas microscopes, allowing measurements of many-body systems with larger occupation number.

Furthermore, the quantum gas magnifier can be employed to access coherence properties with high spatial resolution, for example by applying masks in Fourier space^35^ or by magnification of interference phenomena like Talbot revivals^36^ (see Supplementary Information). We also expect that the sub-lattice spacing resolution would allow band-resolved studies of multi-band systems.

## Methods

### Optical lattice setup

Our optical lattice setup consists of three running waves of wave vector **k**_*i*_ with |**k**_*i*_| = 2π/*λ* intersecting under an angle of 120°. Depending on the polarization of the beams we obtain either a triangular lattice (linear polarization perpendicular to the lattice plane), a honeycomb lattice (linear polarization in plane)^37^ or a boron nitride lattice (suitable elliptical polarization of the lattice beams^38^ as in this work or using spin-dependent light shifts^39^).

The resulting potential can be written as

$$\begin{array}{c}{V}_{{\rm{lat2D}}}({\bf{r}})=\sum _{i > j}\sqrt{{V}_{{\rm{lat}}}^{(i)}{V}_{{\rm{lat}}}^{(j)}}\\ \,\times [{\cos }^{2}(\theta )\,\cos (({{\bf{k}}}_{i}-{{\bf{k}}}_{j}){\bf{r}}+{\alpha }_{i}-{\alpha }_{j})-2{\sin }^{2}(\theta )\,\cos (({{\bf{k}}}_{i}-{{\bf{k}}}_{j}){\bf{r}})]\end{array}$$where the ${V}_{{\rm{lat}}}^{(i)}$ are proportional to the intensities of the lattice beams. *θ* is the angle of the polarization (long half axis) with respect to the lattice plane, *α*_*i*_ is the relative phase between the s and p components of the polarization for beam *i*. We neglected the phases of the beams with respect to each other because they only result in a global shift of the lattice. If we just name a single lattice depth, then all ${V}_{{\rm{lat}}}^{(i)}$ are equal. The boron nitride lattice in Fig. 3 uses *θ* = 9° and *α* = (0,120°,240°) yielding an energy offset between the A and B sublattice quantified by the tight-binding parameter *Δ*_AB_ (ref. ^38^). Note that the triangular lattice has a much larger barrier between nearest neighbours than the honeycomb or boron nitride lattice for the same laser intensities^40^.

### Read-out of lattice site populations

**Extended Data Fig. 1:**
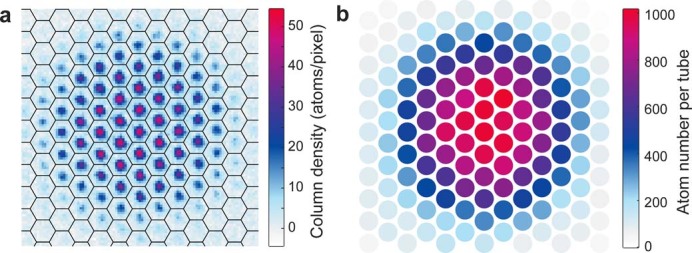
On-site population computation. **a**, Raw density distribution of a typical image. **b**, On-site populations determined from the image in **a** using the Wigner-Seitz masks plotted as solid lines in **a**.

For several experiments only the total population of the lattice sites is of interest. We extract these by first fitting a triangular lattice to the data and subsequently summing up the signal in the Wigner–Seitz cells around the individual sites as explained in the following. The lattice constant *a*_lat_ in pixels is determined by integrating the density of individual images along a real space lattice vector yielding a one-dimensional profile with lattice constant *a*_1D_, which is obtained from a fit with the heuristic function *A*exp(−(*x* − *x*_0_)^2^/(2*σ*^2^))(cos(π*x*/*a*_1D_ + *ϕ*)^2^ + *Δ*). Finally, the lattice constant is deduced from the average fit parameter from two different such directions as ${a}_{{\rm{lat}}}=2{a}_{1{\rm{D}}}/\sqrt{3}$. Next, the spatial phase of the lattice is determined by multiplying the image with a mask that removes the signal from pixels at a certain radius around the sites of a triangular lattice with the lattice constant determined beforehand. The phase of this mask is varied and the configuration minimizing the remaining density is considered the lattice phase. The final step is to determine the population of each lattice site by summing over the Wigner–Seitz cell around the lattice site. To minimize discretization errors the pixels of the camera are subdivided such that the radius of the cell is about ten subpixels. For an example image with non-discretized Wigner–Seitz masks see Extended Data Fig. 1.

For the lattices with two-atomic basis we slightly adjust the algorithm for lattice phase determination by maximizing the density which is not masked thus locating the centres of the honeycombs.

### Lattice phase drifts

For our hexagonal lattice setup composed of three laser beams in two dimensions, phase shifts of the lattice beams only lead to a translation of the whole lattice potential, but not to a change of the lattice geometry^41^. We verify that such phase drifts are not a problem on the time scale of the experiments presented here by measuring the position drift of the atomic cloud’s centre of mass in a very deep optical lattice. We find that the cloud position moves and scatters by less than one lattice site peak-to-peak within 6 s hold time. We checked in a previous set of measurements where we deliberately move the lattice, that the lattice is deep enough to be able to drag the atoms along. Shot-to-shot lattice drifts exceed one lattice site (cycle time of 30 s).

Our characterization of the slow phase drifts is compatible with recent direct measurements of triangular lattices using quantum gas microscopes^42,43^. The drifts can be further reduced to one lattice site per minute in a setup with a single, refolded lattice beam^43^. In our case, the three beams go through separate optical fibres, a setup in which phase locks have been implemented to stabilize the phase^37^. From our characterization, we conclude that a phase lock is not necessary for the measurements presented here. The random lattice phase between individual images can be easily taken into account by identifying the phase. For data evaluation in the main text, we determine the lattice position for every experimental image via a fit routine as described above. Note that the envelope of the atomic density is given by the position of the magnetic trap and is therefore not affected by lattice phase drifts.

### Bimodal fits of density profiles

The lattice-gas profiles can be described by a bimodal model. Since we are considering the on-site populations only, the presence of the lattice can be included by a renormalization of the interaction constant^44^ *g*_eff_ = *g* × *A*_WS_/(2π*σ*^2^) and otherwise using a continuum formalism. Here, *A*_WS_ is the area of the Wigner–Seitz cell, *σ* the on-site radial oscillator length and *g* = 4π*ħ*^2^*a*_sc_/*m* the interaction constant, computed from the scattering length *a*_sc_ ≈ 100 Bohr radii and the mass *m* = 87 u. The on-site radial oscillator length is computed as $\sigma =\sqrt{\hbar /(m{\omega }_{{\rm{onsite}}})}$ from the lattice depth using $\hbar {\omega }_{{\rm{onsite}}}=3\sqrt{2{V}_{{\rm{lat}}}/{E}_{{\rm{rec}}}}{E}_{{\rm{rec}}}$. The data in Fig. 2 is taken with a lattice depth of *V*_lat_ = 1*E*_rec_.

The condensed atoms are described by a 3D Thomas–Fermi profile integrated along line of sight,

1 $${n}_{{\rm{BEC}}}(x,y)=\int {\rm{d}}{z}\frac{15}{8{\rm{\pi }}}\frac{{N}_{{\rm{BEC}}}}{{{R}}_{\rho }^{2}{{R}}_{{z}}}\left(1-\frac{\rho {(x,y)}^{2}}{{{R}}_{\rho }^{2}}-\frac{{{z}}^{2}}{{{R}}_{{z}}^{2}}\right).$$

The fit parameters here are the centre of the cloud *x*_0_, *y*_0_ resulting in *ρ*(*x*, *y*)^2^ = (*x* − *x*_0_)^2^ + (*y* − *y*_0_)^2^, the in-plane Thomas–Fermi radius *R*_*ρ*_ from which the out-of-plane radius *R*_*z*_ is deduced via a computed aspect ratio, and the number of atoms in the BEC *N*_BEC_. In fact, only for the lowest evaporation frequency, where the BEC is very distinct from the thermal part, *N*_BEC_ and *R*_*ρ*_ are fitted independently. For all other fits we compute the Thomas–Fermi radius from the number of condensed atoms using the expected scaling *R*_*ρ*_ = *γN*_BEC_^1/5^ with *γ* determined as its mean value from the fits at lowest evaporation frequency. We obtain *γ* = 0.354 µm, which agrees excellently with the expected value *γ*_theo_ = 0.352 µm obtained from^45^

2 $${\gamma }_{{\rm{theo}}}={15}^{1/5}\sqrt{\frac{\hbar \bar{\omega }}{m{\omega }_{{\rm{system}}}^{2}}}{\left(\frac{{g}_{{\rm{eff}}}}{g}\frac{{a}_{{\rm{sc}}}}{\bar{a}}\right)}^{1/5},$$

supporting the validity of the approximations made. Here *ω*_system_ = 2π × 305 Hz, $\bar{\omega }$ =  (*ω*_system_^2^*ω*_*z*_)^1/3^, *ω*_*z*_ = 2π × 29 Hz and $\bar{a}=\sqrt{\hbar /(m\bar{\omega })}$.

**Extended Data Fig. 2:**
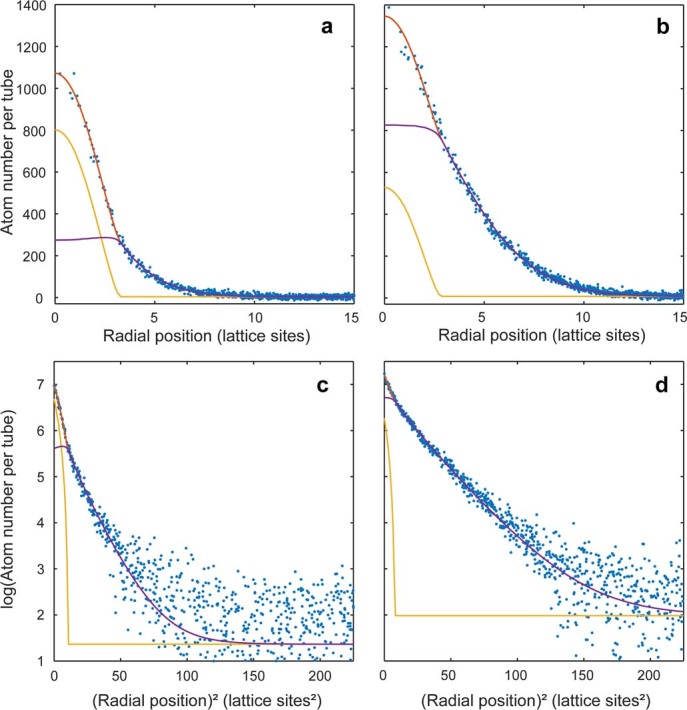
Density as a function of radial position. **a**, **b**, Density of partially condensed BECs in a lattice as function of radial position as shown in Fig. 2c, d. The examples are prepared by end points of the radio frequency evaporation of 85 kHz (**a**) and 105 kHz (**b**) and a hold time at the final evaporation frequency of 1 ms. **c**, **d**, Same data as in **a** and **b** plotted with natural logarithmic *y* axis and quadratic *x* axis resulting in the Gaussian wings appearing as a straight line. Source data

The thermal density distribution is described in a semi-ideal approach, that is, as an ideal gas in a potential *V*(*x*) = *V*_trap_(*x*) + *V*_BEC_(*x*) given by the external trap *V*_trap_(*x*) and the repulsion from the condensed atoms *V*_BEC_(*x*) = 2 *g*_eff_*n*_BEC_(*x*). In semi-classical approximation the ideal Bose gas density distribution is given by^45^

3 $${n}_{{\rm{th}}}(x)={g}_{3/2}(\exp (-\beta (V(x)-\mu )))/{\lambda }_{{T}}^{3}$$

with ${g}_{n}(x)=\sum _{i > 0}{x}^{i}/{i}^{n}$ and ${\lambda }_{{T}}=\hbar \sqrt{2{\rm{\pi }}/(m{k}_{{\rm{B}}}{T})}$. Additionally, we allow for a small offset that we subtract when determining atom numbers. The fit is performed on the 2D density distribution and both the data and the fit function are subsequently plotted as a function of radial position. Extended Data Fig. 2 shows the data from Fig. 2c, d of the manuscript along with a plot of the logarithm of the density versus the square of the radius, which yields a straight line in the thermal wings. This plot shows the excellent agreement between data and fit and also makes the change of the slope at the onset of the BEC fraction more visible.

### Interaction shift and finite size shift

Interactions are known to shift the critical temperature for the BEC transition with a sign depending on the trapping geometry. For a 3D harmonic trap in mean field approximation the shift is negative and given by^20,45^

4 $$\Delta {T}_{{\rm{c}}}/{T}_{{\rm{c}}}\approx -1.33\frac{{g}_{{\rm{eff}}}}{g}\frac{{a}_{{\rm{sc}}}}{\bar{a}}{N}^{1/6}$$

predicting a shift of about −0.24 for the typical atom number of the condensed samples of *N* = 5 × 10^4^, which is larger than the measured shift of −0.099(4). However, for interactions of this strength the mean-field approximation overestimates the shift^22^. Note that we are not aware of a prediction for our 2D–3D crossover geometry of an array of tubes. Our measurements thus set a benchmark for future theoretical studies on the interesting setting of Josephson junction arrays.

We also recall the prediction for the finite size shift of the critical temperature for a 3D harmonic trap. For an anisotropic harmonic trap with trap frequencies *ω*_*x*_, *ω*_*y*_, *ω*_*z*_ and their geometric mean $\bar{\omega }={({\omega }_{x}{\omega }_{y}{\omega }_{z})}^{1/3}$ and arithmetic mean *ω*_m_ = (*ω*_*x*_ + *ω*_*y*_ + *ω*_*z*_)/3, the shift is given by^20,45^.

5 $$\Delta {T}_{{\rm{c}}}/{T}_{{\rm{c}}}\approx -0.73\frac{{\omega }_{{\rm{m}}}}{\bar{\omega }}{N}^{-1/3}.$$

With our trapping frequencies of 2π × (305, 305, 29) Hz, the anisotropy factor is ${\omega }_{{\rm{m}}}/\bar{\omega }=1.53$ and the expected shift is −0.03 for our atom number of *N* ≈ 5 × 10^4^, that is, much smaller than observed. Note that both interactions and finite size effects can contribute to the shift.

The observed smoothing over a range of almost 0.2 in rescaled temperature is only expected for much smaller atom numbers in the case of a 3D harmonic trap^46^. We therefore conclude that finite size effects are strongly enhanced in our 2D–3D crossover geometry of an array of tubes. We have verified that the small condensate fractions involved in the smoothened transition do not arise from fit artefacts of the bimodal profile to the density profiles. The good agreement with the curve for the visibility shown in Extended Data Fig. 4 is further evidence that the signal is physical and demands for further theoretical studies.

### Theoretical description of the density of states

**Extended Data Fig. 3:**
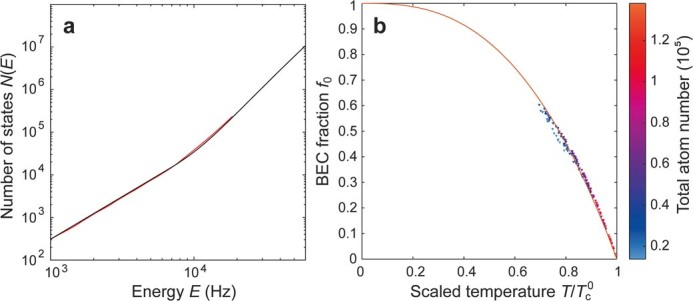
Numerical evaluation of the density of states. **a**, Number of states *N*(*E*) up to energy *E* for a triangular lattice of tubes with tight external confinement: the analytical approximation of equation (8) (black curve) reproduces the numerical diagonalization of the system (red points). The crossover between *α* = 2 and *α* = 3 in the relevant energy range can be seen in the slope change in the log–log plot. **b**, Theoretical values of *f*_0_ for the experimentally accessed parameters computed using the analytical approximation (symbols). The orange curve is a fit of the form ${f}_{0}=1-{(T/{T}_{{\rm{c}}}^{0})}^{\alpha }$ as explained in the main text. Source data

We compare our data of the thermal-to-BEC phase transition to non-interacting calculations based on the density of states. To this end we compute the Hamiltonian matrix for our trap in position basis and diagonalize it. In the numerical spectrum we clearly recognize a crossover between two power laws as a slope change in the log–log plot of Extended Data Fig. 3a. The asymptotes of this crossover can be understood using analytical considerations.

The high energy limit coincides with the well-known spectrum of a 3D harmonic trap resulting in

6 $$N(E)=\frac{1}{6}{\left(\frac{E}{\hbar \bar{\omega }}\right)}^{3}$$

states up to energy *E*. This is due to the fact that the gaps between higher bands are negligible compared to the band widths. So we have to count separately the first band states and harmonic oscillator-like states.

For energies *E* < *Δ*_g_, where *Δ*_g_ is the first bandgap, only states of the first kind are relevant. Here, the tunnel coupling *J* = *h* × ·12 Hz is negligible compared to the offset introduced by the external trap, which is *Δ* = 1/2*mω*_syst_^2^*a*_l*at*_^2^ = *h* × 200 Hz for a site in the centre compared to a nearest neighbour. Hence the spectrum is given by

7 $${E}_{ijk}=1/2m{\omega }_{{\rm{syst}}}^{2}{r}_{ij}^{2}+(k+1/2)\hbar {\omega }_{{\rm{z}}}$$

with *r*_*ij*_ being the distance of the lattice site indexed *ij* from the trap centre and *k* is the index for the *z* direction. A lengthy calculation leads to *N*(*E*) = (*E*/*E*_0_)^2^ with ${E}_{0}=\sqrt{\hbar {A}_{{\rm{WS}}}m{\bar{\omega }}^{3}/{\rm{\pi }}}=h\times 57$ Hz.

We can therefore find an approximation of the numerical result by the Ansatz

8 $$N(E)={\left(\frac{E}{{E}_{0}}\right)}^{2}+\,{\rm{\max }}\left(\frac{1}{6}{\left(\frac{E-\hbar {{\Delta }}_{{\rm{g}}}}{\hbar \bar{\omega }}\right)}^{3},0\right)$$

where *Δ*_g_ is obtained from a simulation without external trap. The crossover between the two power laws appears here at the band gap *Δ*_g_, because the higher bandgaps are small and the lattice can be neglected at higher energies. This analytical model fits very well to the exact diagonalization up to the numerically accessible energies (Extended Data Fig. 3a) while asymptotically reaching the known analytic limit of equation (6) for high energies.

Now we turn to the detailed derivation of the theory curve for a non-interacting system (light-blue line in Fig. 2f). From *N*(*E*) we obtain the density of states *g*(*E*) = d*N*/d*E*, which in turn allows to numerically compute the critical temperature ${T}_{{\rm{c}}}^{0}(N)$ from

9 $$N=\int {\rm{d}}Eg(E)/[\exp (E/({k}_{{\rm{B}}}{T}_{{\rm{c}}}^{0}))-1],$$

that is, ${T}_{{\rm{c}}}^{0}(N)$ is the temperature yielding exactly *N* excited atoms for chemical potential *μ* = 0. The fraction of condensed atoms for a given temperature $T < {T}_{{\rm{c}}}^{0}$ can be computed by first evaluating the number of excited atoms as

10 $${N}_{{\rm{exc}}}=\int {\rm{d}}Eg(E)/[\exp (E/({k}_{{\rm{B}}}T))-1]$$

and then inferring *f*_0_ = (*N* − *N*_exc_)/*N*. Following these steps we can compute ${T}_{{\rm{c}}}^{0}$ and *f*_0_ for every experimental data point from its measured particle number and temperature. The resulting theoretical values are plotted in Extended Data Fig. 3b. We find that these values can be approximated by ${f}_{0}=1-{(T/{T}_{{\rm{c}}}^{0})}^{\alpha }$ as obtained by assuming the density of states $g(E)={C}_{\alpha }{E}^{\alpha -1}$ to be a power law^45^. Fitting the theoretical results for ${f}_{0}(T/{T}_{{\rm{c}}}^{0})$ with *α* as the fit parameter yields *α* = 2.69(1). The corresponding fit shown in Extended Data Fig. 3b is the same line as the light-blue line in Fig. 2f (Extended Data Fig. 3b).

### Comparison to ToF data

**Extended Data Fig. 4:**
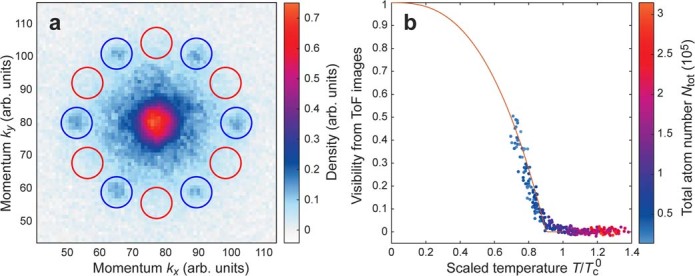
Visibility across the BEC phase transition. **a**, Density after time of flight for the parameters of Fig. 2a in the main text showing the momentum space density with Bragg peaks reflecting the coherence in the system. The visibility is computed as the difference of the density in the blue circles and red circles compared to their sum. **b**, Visibility of the time-of-flight images of the lattice gas as a function of the reduced temperature $T/{T}_{{\rm{c}}}^{0}$ where ${T}_{{\rm{c}}}^{0}$ is the scaling temperature computed from the corresponding real space images in Fig. 2. The line is the fit to the experimental condensate fractions *f*_0_ from the main text for reference. Source data

For comparison, we also take momentum space images from ToF expansion at the same parameters and evaluate their visibility^47^, which is a measure of coherence in the system. We use circular masks around the Bragg peaks (Extended Data Fig. 4a). The radius is determined by fitting the ToF data by a central bimodal distribution

11 $$n({\bf{k}};\sigma ,{k}_{{\rm{R}}},{n}_{0,{\rm{G}}},{n}_{0,{\rm{P}}},{{\bf{k}}}_{0})={n}_{0,{\rm{G}}}\exp (-{({\bf{k}}-{{\bf{k}}}_{0})}^{2}/(2{\sigma }^{2}))+{n}_{0,{\rm{P}}}\,{\rm{\max }}(1-{({\bf{k}}-{{\bf{k}}}_{0})}^{2}/{k}_{{\rm{R}}}^{2},0)$$

and a set of six inverse parabola *n*(*k*; *k*_R_, *n*_0,P_, *k*_0_) = *n*_0,P_max(1 − (*k* − *k*_0_)2/*k*_R_^2^, 0) spaced by a reciprocal lattice vector from the centre, resulting in the complete fit function reading

12 $$\begin{array}{c}n({\bf{k}};\sigma ,{k}_{{\rm{R}},c},{k}_{{\rm{R}},{\rm{Bragg}}},{n}_{0,{\rm{G}}},{n}_{0,{\rm{P}},c},{n}_{0,{\rm{P}},{\rm{Bragg}}},{{\bf{k}}}_{0},{k}_{{\rm{reci}}})\\ \,=n({\bf{k}};\sigma ,{k}_{{\rm{R}},{\rm{c}}},{n}_{0,{\rm{G}}},{n}_{0,{\rm{P}},c},{{\bf{k}}}_{0})+\mathop{\sum }\limits_{j=1}^{6}n({\bf{k}};{k}_{{\rm{R}},{\rm{Bragg}}},{n}_{0,{\rm{P}},{\rm{Bragg}}},{{\bf{k}}}_{0}\\ \,+{k}_{{\rm{reci}}}(\cos \,j{\rm{\pi }}/3,\,\sin \,j{\rm{\pi }}/3))\end{array}$$

where the variables separated by a semicolon are the fit parameters, the parameter *k*_R,Bragg_ is used for the radius and the parameters *k*_0_) and *k*_reci_ for the position of the circular masks. We plot the visibility as a function of $T/{T}_{{\rm{c}}}^{0}$ as obtained from the corresponding real space data (Extended Data Fig. 4b). We plot the theory curve for the condensate fraction as a guide to the eye. This comparison shows that the real-space and momentum-space images give a compatible description of the system.

The visibility and the condensate fraction vanish for the same temperatures (see Fig. 2f and Extended Data Fig. 4). This is in contrast to 3D optical lattices around unit filling, where a finite visibility also for the case of vanishing condensate fraction is observed^18,19^. In these experiments the critical temperatures are much smaller, of the order of a few tunnelling energies, and thus low-energy states that are not the ground state but still have short range phase-coherence are substantially populated yielding a finite visibility above the critical temperature. For our experimental temperatures of a few hundred tunnelling energies no other state than the ground state gets substantially populated.

### Details on magnetic resonance addressing

In order to engineer the density distributions shown in Fig. 3, we used a trap frequency of *ω*_addressing_/2π = 543 Hz for the first five images, of *ω*_addressing_/2π = 658 Hz for the last image and different trap shifts and RF sequences. By shifting the magnetic trap perpendicularly to a real-space lattice vector by around 14 µm, corresponding to approximately twice the system diameter, the curvature of the equipotential lines becomes negligible and the density patterns created byaddressing exhibit straight edges. In Fig. 3 the trap centre resonance frequency is *ω*_c_/2π = 108 kHz for all images, except the last one of panel b where it is *ω*_c_/2π = 67 kHz. The trap is shifted by 14.1 µm for the first and third image, by 15.7 µm for the second image and not shifted for the fourth to sixth image, but always shifted back to the position of the atoms before imaging. For the third image a constant RF pulse of 360 kHz is turned on for 200 ms. For the first image, an RF ramp from 360 to 290 kHz is used, leading to the depletion of all lattice sites from the centre of the cloud towards the centre of the shifted magnetic trap. Here, for the same RF ramp time (200 ms) we ramp over a wider range and therefore have to compensate the reduced time by which the resonance condition is met at each position by increasing the RF amplitude. In all protocols, Fourier broadening is negligible. Lattice phase fluctuations from shot to shot lead to one or two partially depleted rows in most images. The second image in Fig. 3 is created by applying two RF ramps. In this case the trap was shifted further to the side resulting in a higher energy difference to the target *F* = 2, *m*_F_ = 1 state and thus we used ramps from 420 to 486 kHz and from 494 to 540 kHz with 200 ms each to target all sites except for the centre line. For the fourth to sixth image 100 ms were used as the RF duration. In the fourth image the outer wings of the distribution are cut via a RF ramp from 150 to 110 kHz. In the followingimages only a single frequency very close to the respective *ω*_c_, 108.5 and 67.2 kHz, is used to address a ring or a single lattice site. The third and fifth image also visualize the second difference between addressing with and without shifting the magnetic trap: the slope grows linearly from the centre, which leads to sharper resonances for shifted systems.

### Modelling of thermal hopping

The Arrhenius law is often used to describe chemical reaction rates, but also to model thermal hopping of continuously laser-cooled atoms in very deep optical lattices^48^. Here we use it to model the thermal hopping of ultracold atoms in our two-dimensional lattice. In contrast to quantum mechanical tunnelling through the barrier between two lattice sites, thermal hopping refers to motion that is activated thermally when the thermal energy allows to overcome the barrier. To good approximation, the activation energy for a hopping event can be identified with the potential barrier in the lattice, which is *V*_B_ = 9*V*_lat_ in our triangular lattice convention.

The Arrhenius law describes the hopping rate *Γ*_h_ as the product of an attempt rate *Γ*_a_ and the probability *P*(*E* > *V*_B_) to sample an energy *E* above the barrier *V*_B_ in the thermal distribution. The hopping rate can then be written as

13 $${{\Gamma }}_{{\rm{h}}}\approx {{\Gamma }}_{{\rm{a}}}P(E > {V}_{{\rm{B}}})={{\Gamma }}_{{\rm{a}}}({\int }_{{V}_{{\rm{B}}}}^{{\rm{\infty }}}\exp (-E/{k}_{{\rm{B}}}T){\rm{d}}E)/({k}_{{\rm{B}}}T).$$

To include quantum tunnelling, we add an offset rate, resulting in

14 $${{\Gamma }}_{{\rm{h}}}\approx {{\Gamma }}_{{\rm{a}}}\,\exp (-{V}_{{\rm{B}}}/{k}_{{\rm{B}}}T)+{{\Gamma }}_{0}.$$

In Fig. 3, we model the temperature-dependent thermalization rate by the modified Arrhenius law of equation (14) and extract an activation barrier of *V*_B_ = *k*_B_ × 2.4(6) μK and an attempt rate of *Γ*_a_ = 52(44) Hz as well as an offset rate of *Γ*_0_ = 0.23(8) Hz, which we attribute to quantum tunnelling in higher bands. The barrier height for the calibrated lattice depth of *V*_lat_ = 3*E*_r_ is *V*_B_/*k*_B_ = 2.6 μK. We note that in contrast to quantum tunnelling, for thermal hopping the atoms can move over long distances in single hopping events. This enables the large-scale mass transport in Fig. 3 within few hopping events.

### Modelling of nanoscale dynamics

We describe here the numerical simulations shown in Fig. 4c. The simulations start with the ground state of the periodic potential with initial optical lattice beam intensities *I*_2_, *I*_3_ = *I*_1_. At time *t* = 0, *I*_2_ and *I*_3_ are set to 0.5*I*_1_; the intensities change on the intensity lock time scale of about 20 µs. For every time step (5 µs) we diagonalize the Hamiltonian in plane-wave basis of the instantaneous periodic potential and let the state evolve according to the instantaneous eigenstates and eigenvalues. Because the dimers are decoupled from each other, the bands are completely flat and all quasi-momenta are equivalent and we perform the calculations at the Γ point in the Brillouin zone. After the quench, 99.5% of the probability distribution of the time-evolved state is found to lie in the lowest six bands, demonstrating that the dynamics features interference between the two *s* bands and four *p* bands, the latter being the smallest in-plane excitations within a lattice site.

The extracted atomic distribution in a cut of 65 nm width is plotted in Fig. 4c (left). In Fig. 4c (middle) the distribution is convoluted with a Gauss filter of 76 nm width, and summed with an offset, for comparison with the experimental data in Fig. 4c (right). The lattice depth used in the theory (32*E*_rec_; note that the tunnel barriers are much smaller in a honeycomb lattice compared to a triangular lattice of the same total depth) is calibrated from the comparison with the experiment. The external trap is not included in the analysis, because experimentally we don’t see any dependence of the dynamics on the position of the dimer with respect to the trap centre.

## Online content

Any methods, additional references, Nature Research reporting summaries, source data, extended data, supplementary information, acknowledgements, peer review information; details of author contributions and competing interests; and statements of data and code availability are available at 10.1038/s41586-021-04011-2.

## Supplementary information

Supplementary Information Supplementary sections (I)–(III), including Supplementary Figs. 1–5, and additional references.

## Data Availability

All data files are available from the corresponding author on request. Source data are provided with this paper.

## Source data

Source Data Fig. 2
Source Data Fig. 3
Source Data Fig. 4
Source Data Extended Data Fig. 2
Source Data Extended Data Fig. 3
Source Data Extended Data Fig. 4
