# Supplementary material for: Quantum gas magnifier for sub-lattice-resolved imaging of 3D quantum systems
Source: Nature. 2021 Nov 24;599(7886):571–5. doi: 10.1038/s41586-021-04011-2 (PMC8612934; doi:10.1038/s41586-021-04011-2)
Supplement: Supplementary file 1 — Supplementary sections (I)–(III), including Supplementary Figs. 1–5, and additional references. [file 41586_2021_4011_MOESM1_ESM.pdf]

nature portfolio

https://doi.org/10.1038/s41586-021-04011-2

Supplementary information

Quantum gas magnifier for sub-lattice-resolved imaging of 3D quantum systems

In the format provided by the authors and unedited

Supplementary Information for:
Quantum gas magnifier for sub-lattice-resolved imaging of 3D quantum systems

Luca Asteria,[1] Henrik P. Zahn,[1] Marcel N. Kosch,[1] Klaus Sengstock,[1,2,3,*] and Christof Weitenberg[1,2]
[1] *Institut für Laserphysik, Universität Hamburg, 22761 Hamburg, Germany*
[2] *The Hamburg Centre for Ultrafast Imaging, 22761 Hamburg, Germany*
[3] *Zentrum für Optische Quantentechnologien, Universität Hamburg, 22761 Hamburg, Germany*

CONTENTS

I. Matter wave imaging theory 1
A. Focusing condition beyond the far-field limit 1
B. Magnified imaging of the momentum distribution 2
C. Invariance to time-dependent parameters 2
D. Derivation in the Schrödinger picture 3

II. Characterization of the quantum gas magnifier 3
A. Imaging resolution 3
B. Discussion of matter wave aberrations 5
C. Discussion of interaction effects 6

III. Discussion of future directions 7
A. Prospects for reaching single-atom sensitivity 7
B. Prospects for spin-resolved detection 8
C. Prospects for 3D imaging 8
D. Local coherence measurements via Talbot interference 8

References 9

I. MATTER WAVE IMAGING THEORY

A. Focusing condition beyond the far-field limit

In the following, we want to derive the focusing condition for the matter wave optics imaging composed of a quarter period evolution in a harmonic oscillator (HO) trap and a time-of-flight expansion. In particular, we prove that exact imaging can be obtained without having to reach the far field limit of the time-of-flight expansion by adjusting the evolution time in the harmonic trap. We give the derivation for the one-dimensional (1D) case.

We solve the Hamilton equations for the time-dependent $\tilde{x} = x\sqrt{\frac{m\omega}{\hbar}}$ and $\tilde{p} = \frac{p}{\sqrt{\hbar m\omega}}$ operators (in the natural harmonic oscillator units of the harmonic trap with trapping frequency ω and mass m) in the Heisenberg representation:

$$\partial_t \begin{pmatrix} \tilde{x} \\ \tilde{p} \end{pmatrix} = \omega \begin{pmatrix} 0 & 1 \\ -1 & 0 \end{pmatrix} \begin{pmatrix} \tilde{x} \\ \tilde{p} \end{pmatrix} \tag{S1}$$

This gives after a time $t_{\rm ho}$ in the HO:

$$\begin{aligned} \tilde{x}(t_{\rm ho}) &= \cos(\omega t_{\rm ho})\tilde{x}(0) + \sin(\omega t_{\rm ho})\tilde{p}(0) \\ \tilde{p}(t_{\rm ho}) &= \cos(\omega t_{\rm ho})\tilde{p}(0) - \sin(\omega t_{\rm ho})\tilde{x}(0). \end{aligned} \tag{S2}$$

After a time of flight expansion time $t_{\rm tof}$ it becomes:

$$\begin{aligned} &\tilde{x}(t_{\rm ho} + t_{\rm tof}) \\ &\quad= \tilde{x}(t_{\rm ho}) + \tilde{p}(t_{\rm ho})\omega t_{\rm tof} \\ &\quad= \tilde{x}(0)\left[\cos(\omega t_{\rm ho}) - \omega t_{\rm tof}\sin(\omega t_{\rm ho})\right] \\ &\qquad+ \tilde{p}(0)\left[\sin(\omega t_{\rm ho}) + \omega t_{\rm tof}\cos(\omega t_{\rm ho})\right] \\ &\quad= \tilde{x}(0)M\left[\cos(\theta_{\rm tof})\cos(\omega t_{\rm ho}) - \sin(\theta_{\rm tof})\sin(\omega t_{\rm ho})\right] \\ &\qquad+ \tilde{p}(0)M\left[\cos(\theta_{\rm tof})\sin(\omega t_{\rm ho}) + \sin(\theta_{\rm tof})\cos(\omega t_{\rm ho})\right] \end{aligned} \tag{S3}$$

where we introduced the phase space rotation angle $\theta_{\rm tof} = \arctan(\omega t_{\rm tof})$ and $M = \frac{1}{|\cos(\theta_{\rm tof})|} = \sqrt{1 + (\omega t_{\rm tof})^2}$ (Fig. S1). Finally, one can write:

$$\begin{aligned} &\tilde{x}(t_{\rm ho} + t_{\rm tof}) \\ &\quad= M\left[\tilde{x}(0)\cos(\omega t_{\rm ho} + \theta_{\rm tof}) + \tilde{p}(0)\sin(\omega t_{\rm ho} + \theta_{\rm tof})\right] \end{aligned} \tag{S4}$$

The focusing condition is given by

$$\tan(\omega t_{\rm ho}) = -\omega t_{\rm tof} \tag{S5}$$

or equivalently by

$$\theta_{\rm tof} = \arctan(\omega t_{\rm tof}) = -\omega t_{\rm ho} + n\pi, \tag{S6}$$

with n integer.

This condition avoids a mixing of the initial momentum $\tilde{p}(0)$ into the final position $\tilde{x}(t_{\rm ho}+t_{\rm tof})$ and therefore reproduces the initial density distribution without distortion. The magnification of this imaging is given by

$$M = \sqrt{1 + (\omega t_{\rm tof})^2} \approx \omega t_{\rm tof} \tag{S7}$$

or equivalently by

$$M = \frac{1}{|\cos(\omega t_{\rm ho})|}. \tag{S8}$$

Note that we always state M as a positive number although the imaging can be inverting. The focusing condition Eq. (S5) is fulfilled when the evolution time in the harmonic oscillator is close to an odd multiple of a quarter of the oscillation period $T = 2\pi/\omega$. For values of $t_{\rm ho} \sim (1 + 4n)\frac{T}{4}$ values of $\cos(\omega t_{\rm ho} + \theta_{\rm tof})$ are negative and the image would then be inverted, while regular imaging is realized at $t_{\rm ho} \sim (3 + 4n)\frac{T}{4}$. In the far-field limit of the time-of-flight (ToF) expansion $\omega t_{\rm tof} \gg 1$, the magnification simplifies to $M \approx \omega t_{\rm tof}$ and the evolution time in the harmonic trap reduces to $t_{\rm ho} \approx T/4$. For typical magnifications $M > 35$, $t_{\rm ho}$ deviates from $T/4$ by less than 2%.

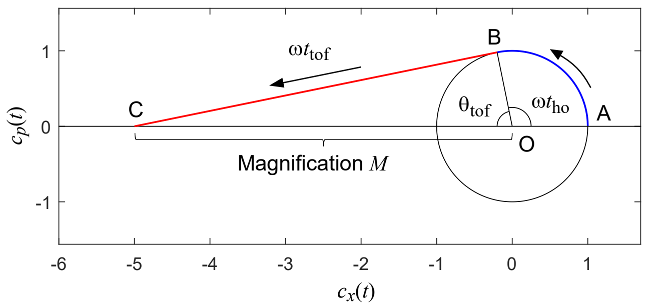

FIG. S1. **Graphical representation of the focusing condition.** Time evolution of the operator $\tilde{x} = c_x(t)\tilde{x}(0) + c_p(t)\tilde{p}(0)$ (with $c_x(t)$,$c_p(t)$ time-dependent coefficients), as a combination of the time-independent operators $\tilde{x}(0), \tilde{p}(0)$. The evolution starts at $A = (1, 0)$ and performs a rotation in the HO for a duration $t_{\rm ho}$. Upon reaching point B, time of flight begins, as described by the straight line BC (since during tof $\tilde{p} = \partial_t\tilde{x} =$ const.) of length $\omega t_{\rm tof}$ (since at all times the velocity in this plane is ω). To get $|c_x| = M$, $c_p = 0$ one sees that $\omega t_{\rm ho} + \theta_{\rm tof} = \pi$ must hold, adding up to the half circle with $\theta_{\rm tof} = \arctan(\omega t_{\rm tof})$, and that $M = \sqrt{1 + (\omega t_{\rm tof})^2}$ as the hypotenuse of the right-angled triangle 0BC. In the far field limit $\omega t_{\rm tof} \to \infty$, $\omega t_{\rm ho} \to \pi/2$ i.e. an exact $T/4$ pulse.

Our large magnifications of up to $M = 93$ are reached via the use of rather large trap frequencies. In the case that larger magnifications are desired, the scheme can easily be adapted. The magnification could be increased via longer time of flight expansion accessible via levitation or by adding an additional time evolution in an anti-confinement after evolution in the harmonic confining potential, in analogy to the proposal for magnification of the momentum distribution in ref.[S1].

B. Magnified imaging of the momentum distribution

In contrast, when choosing $\cot(\omega t_{\rm ho}) = \omega t_{\rm tof}$, one gets $\tilde{x} = M\tilde{p}_0$, i.e. one can measure the momentum distribution without distortion even for finite ToF expansion time $t_{\rm tof}$ but with a magnification arbitrarily tunable in the range between 1 and $\sim \omega t_{\rm tof}$, where 1 is obtained with a pure $T/4$ evolution in the trap[S1,S2] and $\sim \omega t_{\rm tof}$ by a pure time of flight evolution. The advantage of avoiding the far-field approximation has to be balanced with possibly stronger interaction effects during the matter wave optics as discussed in Section II C.

C. Invariance to time-dependent parameters

We provide a proof that distortion free imaging is attainable also in the case where the trap confinement ω or the trap center are time dependent as long as the trap remains harmonic. This is relevant, e.g., for finite switching times of the magnetic trap providing the confinement, which is around 40 µs in our experiments. It is also relevant for magnification protocols during which the trap frequency is ramped up, which can additionally move the trap position due to gravitational sag. For a time dependent trapping frequency $\omega(t)$ one has $\partial_t p = -m\omega(t)^2 x$, which becomes $\partial_t\tilde{p} = -\omega^2/\omega_0\tilde{x}$ after substituting for the dimensionless operators, and ω_0 is the reference time-independent trap frequency entering their definition. Considering also a time dependent trap center shift $c(t)$ of the position operator we can write, in a compact form:

$$\partial_t \begin{pmatrix} \tilde{x} \\ \tilde{p} \end{pmatrix} = \begin{pmatrix} 0 & \omega_0 \\ -\omega^2(t)/\omega_0 & 0 \end{pmatrix} \left[\begin{pmatrix} \tilde{x} \\ \tilde{p} \end{pmatrix} - \begin{pmatrix} c(t) \\ 0 \end{pmatrix} \right]. \quad \text{(S9)}$$

We note that this is still valid in presence of an additional constant force like gravity, since it would just shift the trap center; it has the solution:

$$\begin{pmatrix} \tilde{x} \\ \tilde{p} \end{pmatrix} = U(t) \left[-\int_0^t U^{-1}(t') \begin{pmatrix} c(t') \\ 0 \end{pmatrix} dt' + \begin{pmatrix} \tilde{x}(0) \\ \tilde{p}(0) \end{pmatrix} \right] \quad \text{(S10)}$$

with $U(t)$ (with the matrices in the product ordered from right to left):

$$U(t) = \lim_{\mathrm{d}t \to 0} \prod_{n=0}^{\frac{t}{\mathrm{d}t}} \left[1 + \mathrm{d}t \begin{pmatrix} 0 & \omega_0 \\ -\omega^2(n\mathrm{d}t)/\omega_0 & 0 \end{pmatrix} \right] \quad \text{(S11)}$$

The first term of the sum in Eq. (S10) just shifts $\tilde{x}$ by a (time-dependent) real number (the image would be displaced). The evolution restricted to the space spanned by the linear combinations of $\tilde{x}(0)$ and $\tilde{p}(0)$ is then described just by

$$\partial_t \begin{pmatrix} \tilde{x} \\ \tilde{p} \end{pmatrix} = \begin{pmatrix} 0 & \omega_0 \\ -\omega^2(t)/\omega_0 & 0 \end{pmatrix} \begin{pmatrix} \tilde{x} \\ \tilde{p} \end{pmatrix}, \quad \text{(S12)}$$

which is solved by

$$\begin{pmatrix} \tilde{x} \\ \tilde{p} \end{pmatrix} = U(t) \begin{pmatrix} \tilde{x}(0) \\ \tilde{p}(0) \end{pmatrix}. \quad \text{(S13)}$$

The matrix $\begin{pmatrix} 0 & \omega_0 \\ -\omega^2(t)/\omega_0 & 0 \end{pmatrix}$ implies a rotation with angular velocity $\partial_t\theta > \min(\omega^2(t)/\omega_0, \omega_0)$ which is always finite as long as $\omega(t)$ remains finite, and the additional time of flight can only rotate vectors $\theta_{\rm tof} < \frac{\pi}{2}$: from continuity it follows that it must exist an optimal time in the HO that ensures zero $\tilde{p}(0)$ component in $\tilde{x}$. In such cases, the expression for the magnification might be involved. The finite switching time of the magnetic trap of 40 µs is about 10% of $T/4$ for the biggest $\omega/2\pi \approx 700$ Hz, resulting in a deviation of similar magnitude of M from the estimate $\omega t_{\rm tof}$. Therefore, we determine the magnification experimentally by comparing the measured lengths of lattice vectors with the expected ones.

D. Derivation in the Schrödinger picture

We provide for completeness the derivation for the magnification in the Schrödinger picture, which is completely equivalent to the Heisenberg's one but it is useful because it describes the evolution of the quantum field operators $\Psi^\dagger(r)$ illustrating that the matter wave optics also reproduces quantum correlations. This possibility will allow for future fundamental studies in single-atom resolved regimes with the quantum gas magnifier.

We make use of the generating function G of the Hermite polynomials $h_n(x)$ given by

$$G(x,g) = e^{-\frac{1}{2}x^2+2xg-g^2} = \sum_n e^{-\frac{1}{2}x^2} h_n(x)\frac{g^n}{n!}. \quad \text{(S14)}$$

Using the operator $\hat{O}_n = (\partial g)^n|_{g=0}$, one gets

$$\hat{O}_n G(x,g) = e^{-\frac{1}{2}x^2} h_n(x) = \psi_n(x) \quad \text{(S15)}$$

with $\psi_n(x)$ being the nth eigenstate of the HO and with x being the spatial coordinate in natural units. Up to a global phase, the time evolution in the HO $U(t_{\text{ho}})$ can be described by

$$U(t_{\text{ho}})G(x,g) = G(x, ge^{-i\omega t_{\text{ho}}}) \quad \text{(S16)}$$

which is proven by checking that ψ_n picks up a phase $\phi_{\text{ho},n} = -n\omega t_{\text{ho}}$:

$$U(t_{\text{ho}})\psi_n(x) = \hat{O}_n G(x, ge^{-i\omega t_{\text{ho}}}) = \psi_n(x)e^{-in\omega t_{\text{ho}}}. \quad \text{(S17)}$$

The time of flight evolution $U(t_{\text{tof}})$ of G can be described by

$$U(t_{\text{tof}})G(x,g) = F^{-1}(\sqrt{2\pi}\int dx' e^{-\frac{1}{2}x'^2+2x'g-g^2} e^{ikx'-ik^2\frac{T}{2}}) \quad \text{(S18)}$$

with $T = \omega t_{\text{tof}}$ and F^{-1} being the inverse Fourier Transform operator. It follows:

$$\begin{aligned} &F^{-1}(\sqrt{2\pi}\int dx' e^{-\frac{1}{2}(x'-2g-ik)^2-\frac{1}{2}k^2-ik^2\frac{T}{2}+2ikg+g^2}) \\ =&F^{-1}(e^{-\frac{1}{2}k^2+g^2-ik^2\frac{T}{2}+2ikg}) \\ =&\sqrt{2\pi}\int dk e^{-\frac{1}{2}(k^2 D-\frac{2ig}{\sqrt{D}}+\frac{ix}{\sqrt{D}})^2+g^2+\frac{1}{2D}(-4g^2-x^2+4xg)} \end{aligned} \quad \text{(S19)}$$

with $D = 1+iT$. One gets, recalling $M = \sqrt{1+T^2}$:

$$\begin{aligned} &= \frac{1}{\sqrt{D}} e^{-\frac{x^2}{2D}+\frac{2xg}{D}+g^2(1-\frac{2}{D})} \\ &= \frac{1}{\sqrt{D}} e^{\frac{x^2}{2}\frac{iT}{1+T^2}} e^{-\frac{x^2}{2}\frac{1}{1+T^2}+\frac{2xg}{1+iT}-\frac{1-iT}{1+iT}g^2} \\ &= \frac{1}{\sqrt{M}} e^{-i\frac{\arctan(T)}{2}+\frac{iT}{2}(\frac{x}{M})^2} G(\frac{x}{M}, g\cdot e^{-i\cdot\arctan(T)}) \end{aligned} \quad \text{(S20)}$$

It follows:

$$\begin{aligned} &U(t_{\text{tof}})\psi_n(x) = \hat{O}_n U(t_{\text{tof}})G(x,g) \\ =&\frac{1}{\sqrt{M}} e^{-i\frac{\arctan(T)}{2}+\frac{iT}{2}(\frac{x}{M})^2} \psi_n(\frac{x}{M}) e^{-in\cdot\arctan(T)} \end{aligned} \quad \text{(S21)}$$

One gets a normalization factor $\frac{1}{\sqrt{M}}$ and a n-independent phase $e^{i\phi(x,T)} = e^{-i\frac{\arctan(T)}{2}+\frac{iT}{2}(\frac{x}{M})^2}$. During time of flight, ψ_n gets magnified by a factor M and picks up a phase $\phi_{\text{tof},n} = -n\cdot\arctan(T)$. The total time evolution of a generic wavefunction $\psi(x) = \sum_n c_n\psi_n(x)$ during the magnification protocol is then:

$$U(t_{\text{tof}})U(t_{\text{ho}})\psi(x) = \frac{1}{\sqrt{M}} e^{i\phi(x,T)} \sum_n c_n \psi_n(\frac{x}{M}) e^{i\phi_n} \quad \text{(S22)}$$

and the focusing condition Eq. (S5) for the magnified imaging can then be obtained by requiring that

$$\phi_n = \phi_{\text{ho},n} + \phi_{\text{tof},n} = -n\cdot[\omega t_{\text{ho}} + \arctan(\omega t_{\text{tof}})] = -n\cdot\pi \quad \text{(S23)}$$

As a consequence, the terms in the superposition with n even (odd), corresponding to states $\psi_n(x)$ symmetric (antisymmetric) w.r.t $x=0$, pick up a phase 0 (π), as if under the action of the parity operator $P\psi(x) = \psi(-x)$; for this reason the distribution is inverted. The phase factor $e^{\frac{iTx^2}{2M^2}}$ means that although density correlations $g_2(r)$ are reproduced via the scaling $g_2(r') = g_2(M\cdot r)$, phase correlations $g_1(r)$ have to be treated with care. This comes from the fact that the protocol presented here simply rescales the real space positions, but not the momentum operator.

II. CHARACTERIZATION OF THE QUANTUM GAS MAGNIFIER

A. Imaging resolution

In order to characterize the matterwave optics and the employed magnetic trap, we study the images for odd multiples of $T/4$, i.e. matterwave relay imaging by adding $T/2$ evolutions, in which all aberrations are amplified and can be better quantified. We define the contrast of the quantum gas magnifier as the integrated strength of the peaks around the reciprocal lattice vectors in the Fourier transformation of the measured densities normalized to the total atom number. Fig. S2a shows how the lattice contrast of the images decreases for these multiples as expected. Fig. S2b shows the contrast around the expected evolution times in the trap t_{ho} corresponding to $(2n+1)T/4$ for $n = 0, ..., 5$. For a larger number of relay imaging steps n, the contrast cannot be recovered and the contrast also gets worse for larger atom numbers. This data is taken after removing the coherence between different tubes by ramping into a deep optical lattice. Without removing coherence, the contrast is lost much faster.

From this data, we also obtain the precise time of maximal contrast, which we plot in Fig. S2c. We find that the time of optimal contrast slightly depends on the direction in which the contrast is evaluated (Fig. S2d) and we attribute this to the graviational sag, which slightly opens the trap along the vertical direction. The resulting ellipticity is only on the order of 1% allowing us to focus the image in x and y direction simultaneously. The exact time of optimal contrast also slightly depends on the atom number as depicted in Fig. S2e,f. We attribute this to a mean field repulsion, which effectively reduces the trap frequency of the external trap.

For a more quantitative measure of the resolution of the quantum gas magnifier, we fit a grid of Gaussians with global $1/\sqrt{e}$ radius σ_{site} to a central cut of the images along the three lattice axes. We repeat this for several magnifications M, i.e. different trapping frequencies, and plot the resulting width versus magnification (Fig. S3a,b). For magnifications above $M = 35$, the lattice sites are well resolved according to the Rayleigh criterion with $\sigma_{\mathrm{site}} < 0.35 a_{\mathrm{lat}}$. The experimental resolution σ_{site} can be reasonably well described as a convolution of the actual size of the wavefunction in the lattice site σ_{wf} multiplied by the magnification M and the optical resolution of the absorption imaging σ_{opt}:

$$\sigma_{\mathrm{site}}(M) = \sqrt{\sigma_{\mathrm{opt}}^2 + (M\sigma_{\mathrm{wf}})^2}. \tag{S24}$$

The brown line in Fig. S3b shows this dependency, yielding $\sigma_{\mathrm{opt}} = 5.2(2)\,\mu\mathrm{m}$, $\sigma_{\mathrm{wf}} = 118(3)\,\mathrm{nm}$.

A more careful analysis should also include interaction effects during the matter wave optics. In order to increase these usually small effects, we repeat the analysis for different atom numbers N and for odd multiples of $T/4$ (Fig. S3c). A more complete description is then given by the heuristic model

$$\begin{aligned}\sigma_{\mathrm{site}}^2(M,N,n) =& \sigma_{\mathrm{opt}}^2 + (M\sigma_{\mathrm{wf}})^2 + ([2n+1]^{p_1} N^{p_2} M\sigma_{\mathrm{int}})^2 \\ &+ ([2n+1]^{p_3}\sigma_{\mathrm{lens}})^2\end{aligned} \tag{S25}$$

with the parameters $\sigma_{\mathrm{opt}} = 5.3(3)\,\mu\mathrm{m}$, $\sigma_{\mathrm{wf}} = 68(24)\,\mathrm{nm}$, $\sigma_{\mathrm{int}} = 4.2(4)\,\mathrm{nm}$, $\sigma_{\mathrm{lens}} = 0.42(46)\,\mu\mathrm{m}$ and the exponents $p_1 = 0.29(5)$, $p_2 = 0.33(5)$ and $p_3 = 1.6(7)$. Here σ_{int} takes into account the broadening due to interactions and σ_{lens} the single particle broadening. Note that the optical resolution σ_{opt} is given here as the $1/\sqrt{e}$-width of the point spread function, which corresponds to a Rayleigh resolution of about $r_0 = (3.83/1.35)\sigma_{\mathrm{opt}} = 15\,\mu\mathrm{m}$. The dashed lines in Fig. S3b show the fit of Eq. (S25) using σ_{lens}, p_1, p_2 and p_3 from Fig. S3c, yielding very similar results: $\sigma_{\mathrm{opt}} = 5.2(1)\,\mu\mathrm{m}$, $\sigma_{\mathrm{wf}} = 75(10)\,\mathrm{nm}$, $\sigma_{\mathrm{int}} = 3.6(3)\,\mathrm{nm}$. The optical resolution also agrees with the simpler fit of Eq. (S24), whereas σ_{wf} is overestimated in that case due to the negligence of interaction effects.

We note that an estimation of the scattering rates based on the formula for classical particles $\Gamma_{\mathrm{s}} = n\sigma_{\mathrm{s}}v$ (with n the particle density, σ_{s} their scattering cross-section and v their mean velocity) yields a scattering

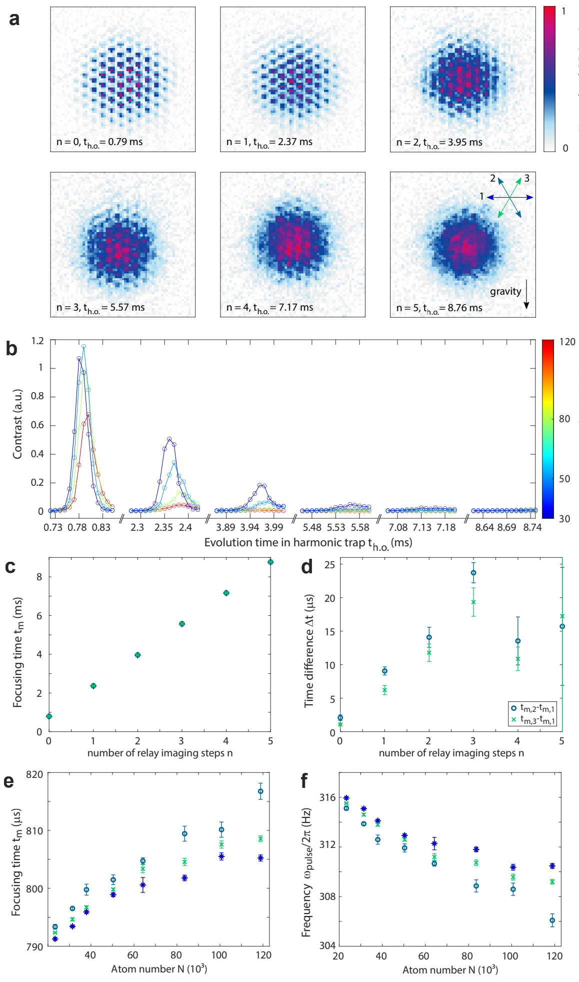

FIG. S2. **Image contrast of the quantum gas magnifier.** **a**, Images of the triangular lattice using increasing numbers of odd multiples in the harmonic trap: $(2n+1)T/4$ for $n = 0, ..., 5$. The average atom number is 30,000 corresponding to the dark blue color in b. **b**, Lattice contrast versus the evolution time t_{ho}. The colors specify the average atom number used in the images. **c**, Focusing times t_m obtained as times of maximal contrast determined by fitting a Gaussian function to every contrast peak of the blue lines from b (blue stars: direction 1, teal circles: direction 2, green crosses: direction 3, for comparison of the directions see inset in a). **d**, Differences between the times of the two 1D lattice along direction 2 and 3 (not perpendicular to gravity) and the 1D lattice along direction 1 (perpendicular to gravity), illustrating the slight ellipticity along gravity. **e**, Times of maximal contrast for $n = 0$ and different atom numbers for the three 1D lattices. **f**, Corresponding effective trap frequencies, the decrease of which we attribute to mean field repulsion. All error bars correspond to the 68% confidence interval.

probability per atom smaller than 1% over the whole $T/4$ evolution, for all of the experiments described here with removal of coherence. For coherent systems with their larger density peaks during the $T/4$ evolution, the number of scattering events can be significantly higher.

The influence of interaction should therefore be

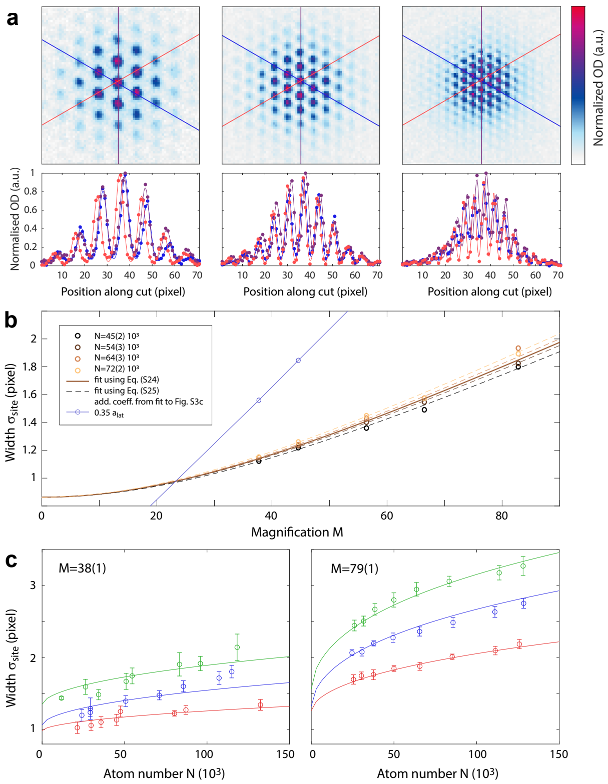

FIG. S3. **Resolution of the quantum gas magnifier.** **a**, Images with varying magnification (from left): $M = 83(1)$, 56(1), 38(1). Beneath are cuts through the most populated rows along the three lattice directions, marked by correspondingly coloured lines in the images. The lines connecting the data points result from fitting a grid of Gaussian functions with a global $1/\sqrt{e}$ radius σ_{site} for every lattice site. **b**, Fitted σ_{site} for five different magnifications and varying atom number. The brown line shows the fit according to Eq. (S24), independent of the atom number. The dashed lines describe the dependency from Eq. (S25), using the parameters σ_{lens} and p_1, p_2 and p_3 from **c**. The lattice sites can be resolved according to the Rayleigh criterion when $\sigma_{\text{site}} < 0.35 a_{\text{lat}}$ (blue line). **c**, Gaussian width σ_{site} from fits to data along cuts in images as in Fig. S2a for different atom numbers, the first three odd multiples of T/4 (red: n=0, blue: n=1, green: n=2) and two magnifications. The two line triplets are from a common fit of Eq. (S25) to the data in both panels in **c**. All error bars correspond to the 68% confidence interval.

thought of via the repulsive mean-field potential, which modifies the effective potential seen be the atoms. Indeed, the effective trapping frequency is reduced by about 2% when increasing the atom number (Fig. S2e,f), which can then be compensated by the proper focusing time. However, the density distribution during the $T/4$ evolution is not harmonic and the anharmonicity can lead to distortions of the image similar to the anharmonicity of the trap itself. Estimates show that the quartic part of the mean field potential for our typical parameters can be of the same order of magnitude, but with opposite sign, as the quartic part of the trap. As an example, we make an estimate for the parameters in (Fig. S3c) with a large magnification $M = 79$ and an atom number $N = 10^5$. We obtain a Gaussian atomic distribution with width $\sigma_{\text{sys}} = 1.45\,\mu$m at the end of the $T/4$ evolution and a corresponding mean-field potential with quartic term of amplitude $15.7\text{Hz}/(\mu m)^4$, comparable to the quartic term of the trap of $-7.2\text{Hz}/(\mu m)^4$ (Compare also with section II B). A more quantitative theoretical analysis of the influence of interactions on the resolution of the quantum gas magnifier is left for future work.

In contrast to the analysis above, the coherence was not removed for the nanoscale dynamics data of Fig. 4 and we therefore expect more interaction effects due to the residual coherence even in the relatively deep lattice. Indeed, we find about 17% of the atoms scattered into a constant background (compare the different color maps in Fig. 4c). These stronger interaction effects might also be related to the more complicated situation of a honeycomb lattice, in which scattering processes at the beginning of the ToF expansion can play a role for spin-mixtures[S3,S4].

B. Discussion of matter wave aberrations

The matter wave optics can also include single particle aberrations, e.g. due to anharmonicity of the matter wave lens. The choice of a magnetic trap for the harmonic oscillator potential allows not only for a very smooth and isotropic trap, but also for smaller anharmonicities than in typical optical traps. The anharmonic (or aspheric) aberration can therefore be said to set a limitation on the usable field of view. For our magnetic trap, such aberrations become visible only for very large magnifications and for large systems or when displacing the cloud relative to the trap center. In order to control and characterize this anharmonicity we image a cloud shifted off-center in a very strong magnetic trap with a trap frequency in the $x - y$ plane of $\omega_{\text{ho}} = 2\pi \cdot 641\,\text{Hz}$, which results from a gradient $B_1 = 1.69 \cdot 10^4\,\text{G/m}$, a trap bottom $B_0 = 0.112\,\text{G}$ and an anticurvature $B_2 = 7.1 \cdot 10^5\,\text{G/m}^2$ (Fig. S4). The trap potential V_{trap} can then be written as

$$V_{\text{trap}}/h = 78.4\,\text{kHz} + 1756\,\text{Hz}(\rho/\mu\text{m})^2 - 7.2\,\text{Hz}(\rho/\mu\text{m})^4 \quad \text{(S26)}$$

where ρ is the distance from the trap center and 78.4kHz is the resonance to the $m_F = 1$ state in the center of the trap. An optical trap with a waist of 16 µm has the same quartic term to quadratic term ratio. This trap has a relatively high trapping frequency and anharmonicity, in general the magnetic trap is much more tunable and for example we could get a trap frequency as high as $2\pi \cdot 200\,\text{Hz}$ with an anharmonicity corresponding to a 135 µm waist optical trap. For suitable parameters of an optical trap with small anharmonicity, intensity requirements for reaching the high trapping frequencies might be an important factor. The small distortion of the im-

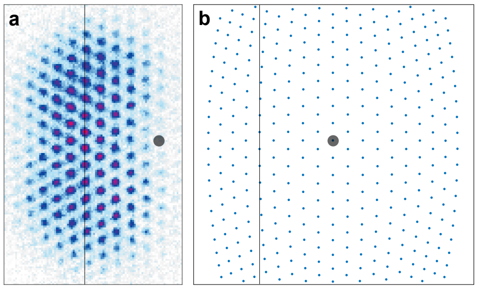

FIG. S4. **Aberrations due to trap anharmonicity.** **a**, Sum of two images with the cloud displaced in different directions. For large trapping frequencies (here $\omega_{\mathrm{ho}} = 2\pi \cdot 641\,\mathrm{Hz}$) and atoms far from the center of the trap ('optical axis', grey circle), the anharmonicity of the trap gives rise to a distortion of the matter wave optics image, directly visible as a distortion of the imaged lattice . **b**, The distortion of the positions can be reconstructed by a simulation of the dynamics of a lattice of classical point particle.

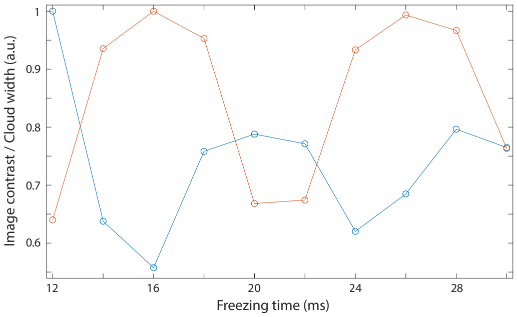

FIG. S5. **Contrast oscillation.** Width of the cloud along the line of sight of the usual imaging direction after the magnification protocol, as a function of the time after the excitation of a breathing mode in the direction perpendicular to the lattice (red curve). Broader clouds have a smaller widths during the first stages of the protocol and hence enhanced interactions. This leads to a smaller contrast of the quantum gas magnifier (blue curve).

age is no limitation when one is interested in the lattice site occupations.

The small effect of aberrations due to anharmonicity or other imperfections in magnetic traps is also supported by the observation in ref.[S6] that the density distribution after a $T/2$ evolution in a harmonic trap is almost identical to the original distribution for the case of a non-interacting two-dimensional (2D) Fermi gas.

C. Discussion of interaction effects

The matter wave optics imaging is initialized by projecting onto a non-interacting system. Quick reduction of the density is achieved here by switching off the optical lattice; other possibilities are a fast release of the transverse confinement in bulk 2D systems or a switching off of the interactions via a Feshbach resonance. For suppressing interaction effects during the matter wave optics, we typically remove the coherence by freezing in a deep lattice of $6\,E_{\mathrm{rec}}$ ($J/h \sim 0.001$Hz) for 12 ms right before the magnification protocol. This avoids the density peaks, which otherwise arise during the $T/4$ evolution due to interference (Talbot revivals for short times and Bragg peaks at the end of the $T/4$ pulse).

For the analysis of the density sector in this article, removing the coherence is unproblematic and interaction effects are then small.

In the experiments corresponding to Fig. 1-3 we remove the coherence with the scheme presented above. For the data presented in Fig. 4, we reduce the coherence by starting in a deep lattice.

When ramping up the lattice intensity for the coherence removal procedure one can excite breathing oscillations along the transverse direction (z-direction), over which the signal is integrated. This can increase the density during the $T/4$ evolution and therefore reduce the contrast due to interaction effects. The resulting oscillation of the contrast (Fig. S5) fits well to a breathing mode for the independent characterization of the transverse trapping frequency of the optical lattice. This analysis shows that the dynamics along the transverse direction is very important for the size of interaction effects. Therefore a protocol, which leads to a rapid expansion in the transverse direction without bringing the cloud out of the depth of focus of the optical imaging system, can be beneficial[S1].

When not exciting breathing modes, there is no significant dynamics during the whole protocol along the z-direction. The residual harmonic confinement in z-direction during $T/4$ is very weak when compared to ω_{pulse} (after switching off the lattice, $\omega_z \sim 2\pi \times 11$ Hz) and can be neglected. The scale of the energies involved in this direction is then set by the initial transverse trap frequency $\omega_z \sim 2\pi \times 29$ Hz which leads, over a time of flight of about ~ 20 ms, just to a small (relative) lengthening of the tubes, initially characterized by a Thomas-Fermi radius of about $30\,\mu$m. We note that the extension in the z direction therefore stays significantly below the depth of focus of the optical imaging of about $800\,\mu$m. Our characterizations provide a benchmark for the capability of the quantum gas magnifier concept and are also relevant for matter wave optics for imaging momentum space. This analysis demonstrates the full control of the quantum gas magnifier even including interaction effects for Rb-atoms and typical magnifications $M = 50 - 90$. For other elements like ^{7}Li or ^{39}K the use of appropriate Feshbach resonances will even allow further extensions of the magnifier as interaction effects can be fully switched off prior to magnification and imaging. Vice versa interaction effects can be studied with the system with careful prior calibration.

Alternatively, interaction effects during the magnification protocol would be sufficiently suppressed for the

small background scattering lengths at low magnetic fields, allowing to combine the use of Feshbach resonances for the initial system of interest with a tight magnetic trap for the matter wave optics.

In addition, we note that while possible interaction effects during the matter wave optics protocol have to be treated with care, the magnification and its resulting low densities completely remove interaction effects during the optical imaging, such as saturation effects at high optical densities[S7] or light-assisted collisions in tight traps. This avoids distortions of the obtained density profile and reduces systematic effects on the absolute atom number calibration of absorption imaging.

III. DISCUSSION OF FUTURE DIRECTIONS

A. Prospects for reaching single-atom sensitivity

The quantum gas magnifier can be extended to single-atom sensitivity as we demonstrate in the following. This is relevant both for studying arrays of tubes in three-dimensional (3D) systems or for imaging single 2D systems, i.e. for reaching strongly-correlated regimes and accessing quantum correlations as in conventional quantum gas microscopes[S8]. The estimate below follows the free-space fluorescence imaging of few atoms demonstrated for ^{87}Rb atoms[S9–S11] and for ^{6}Li atoms[S12]. Alternatively, combining the quantum gas magnifier with the single-atom detection of metastable helium on multi-channel plates[S13] would also allow single-atom sensitivity and could even be devised to include a 3D real-space imaging as already achieved in momentum space (see e.g. ref.[S14]).

In the following, we exemplarily estimate the expected number of photons from a single ^{87}Rb atom of mass m. The recoil velocity from scattering a photon of wavelength $\lambda = 780\,\mathrm{nm}$ is $v_{\mathrm{rec}} = h/(\lambda m) = 5.88\,\mathrm{mm/s}$. The random recoils lead to a random walk with a width $\sigma(\tau) = \frac{1}{3} v_{\mathrm{rec}} R^{1/2} \tau^{3/2}$ after scattering for a time τ with a rate R[S15]. Due to their smaller recoil velocity, heavier atoms allow to scatter more photons before reaching a certain width. For a magnification of $M = 70$, the lattice spacing of the triangular lattice $a_{\mathrm{lat}} = 709\,\mathrm{nm}$ is magnified to $M a_{\mathrm{lat}} = 50\,\mu\mathrm{m}$. We therefore want to restrict the width of the random walk to $\sigma = 15\,\mu\mathrm{m}$, in order to keep the signal from the different lattice sites separated. This choice restricts the scattering time to $\tau = 150\,\mu\mathrm{s}$ for a resonant saturated scattering rate $R = \Gamma/2$ with $\Gamma = 2\pi \cdot 6\,\mathrm{MHz}$, yielding $N = R\tau = 2700$ scattered photons. The number of detected photons crucially depends on the numerical aperture (NA), which dictates the solid angle $\Omega \sim \pi \mathrm{NA}^2$, from which the photons are collected. For a medium high NA of 0.3, the solid angle covers a fraction $\Omega/(4\pi) \sim 2.3\%$ of the unit sphere. Assuming a transmission of the imaging system of 80% and a quantum efficiency of the camera of 75%, this yields 36 detected photons. On an electron multiplying charge-coupled device (EMCCD) camera, an average number of 25 detected photons is sufficient for a signal above the noise level[S12]. This estimate does not include the possibly slightly enhanced scattering into the imaging system due to the dipole radiation pattern for correct choice of the magnetic field direction and polarization. We emphasize that this estimate explicitly uses the matter wave magnification to bring the atoms to large distances of e.g. 50 µm, where the signals from different lattice sites do not overlap. This is in contrast to conventional quantum gas microscopes with an *in situ* detection of atoms at 0.5 µm distances, where a few hundreds to thousands of photons are required for distinguishing the atoms. This magnification and the resulting dilution will also allow imaging systems with several atoms per lattice site by avoiding density-dependent processes such as light-assisted collisions.

Another limitation to the duration of the optical imaging comes from the fact that the atoms are in free fall. At the end of the time-of-flight expansion of duration $t_{\mathrm{tof}} = 25\,\mathrm{ms}$, they have acquired a velocity $v = g t_{\mathrm{tof}} = 0.25\,\mathrm{m/s}$, where $g = 9,81\,\mathrm{m/s}^2$ is the gravitational acceleration. To keep the displacement during the imagine pulse of length t_{im} below a lattice constant $M a_{\mathrm{lat}} = 50\,\mu\mathrm{m}$, it is restricted to $t_{\mathrm{im}} \ll M a_{\mathrm{lat}}/v = \omega_{\mathrm{ho}} a_{\mathrm{lat}}/g = 200\,\mu\mathrm{s}$. This is no limitation to standard absorption imaging, but becomes relevant for reaching large signals per atoms, in particular when the diffusion from photon scattering allows longer imaging times such as for heavy elements. Solutions include a magnetic levitation during ToF, vertical orientation of the imaging, or the use of fluorescence imaging with carefully imbalanced beam intensities to decelerate the atoms.

Even without the gravitational acceleration, the atoms obtain a velocity from the matter wave transformation itself, which is given by $v = n a_{\mathrm{lat}} \omega_{\mathrm{ho}}$ with the initial distance from trap center $n a_{\mathrm{lat}}$. This velocity restricts the imaging time to $t_{\mathrm{im}} \ll t_{\mathrm{tof}}/n$, which could become a limitation for large systems or displacements in the trap with $n \gg 100$. This would still allow larger system sizes than typically achieved in conventional quantum gas microscopes.

We expect that starting from Hubbard-like systems, where the single-atom sensitivity is most relevant, does not introduce particular limitations to the matter-wave optics, because the rapid initial expansion after switching off the lattice will also hold in this case. Let us recall that also time-of-flight images work equally well starting from Mott insulators or superfluids. In particular, working with a single 2D system should reduce interaction effects due to the rapid expansion when switching off the 2D confinement[S1]. As a first test, we have measured the magnified density distribution of a system with an additional z-lattice, which brings the system into a 3D Hubbard regime, and have found no reduction of the contrast of the images.

B. Prospects for spin-resolved detection

While we restrict our measurements to atoms in a single spin state, the scheme can be straight-forwardly extended to a spin-resolved detection of spin-mixtures. This is relevant in particular for the Fermi-Hubbard model, but requires complicated protocols in conventional quantum gas microscopes for the detection of both spin states of a single snapshot[S16,S17]. One possibility is to choose two spin states with the same magnetic-field dependence, which experience the same harmonic confinement during the $T/4$ pulse. A global microwave sweep or Raman coupling, which transfers one of the states to a state with different magnetic field dependence then allows to spatially separate the two spin states during ToF expansion by adding a magnetic field gradient in a Stern-Gerlach configuration. Using a magnetic-field independent state during the $T/4$ pulse would allow to simultaneously image the real-space distribution for one spin state and the momentum-space distribution for the other spin state. When using a spin-independent optical trap for the $T/4$ pulse, one can work with two spin states with different magnetic moment from the beginning and the step of changing the spin state can be omitted. The compatibility of Stern-Gerlach separation with free-space fluorescence imaging was recently demonstrated in ref.[S18].

C. Prospects for 3D imaging

We note that tomographic 3D imaging, e.g. via scanning along the z-direction[S19,S20], should also be applicable to the magnified system. This requires focused matter wave imaging in all three directions, which can be achieved with a fully isotropic trap. The lattice sites are then also separated by typically 50 µm in the imaging direction such that either a suitable depth of focus or spatially resolved optical pumping can be used for imaging the slices separately.

D. Local coherence measurements via Talbot interference

In this section, we discuss how the concept of the quantum gas magnifier – so far used to image real space density – could be extended to the realm of coherent phenomena. The Talbot effect in optics describes the revival of a lattice structure with spatial periodicity $L_{\text{Talbot}} = 2(Ma_{\text{lat}})^2/\lambda$ after transmission through a periodic potential with lattice constant a_{lat}. The Talbot effect manifest itself also with matter waves[S21–S23] and it is based on the fact that a periodic 1D wavefunction can be decomposed in the plane wave basis at multiples of the wavevector $k = 2\pi/a_{\text{lat}}$ with kinetic energies $E_n = n^2(\hbar k)^2/(2m)$ multiple of the same fundamental frequency, whose inverse is the Talbot period T_{Talbot}.

We note that in 2D Talbot revivals also appear in the case of triangular and honeycomb lattice because here the allowed wavevectors are $\mathbf{k}_{n,m} = n\mathbf{k}_a + m\mathbf{k}_b$ with $\mathbf{k}_a = (1,0)$, $\mathbf{k}_b = (-\frac{1}{2}, \frac{\sqrt{3}}{2})$ and n, m integers, with associated kinetic energies $E_{n,m} \propto |\mathbf{k}_{n,m}|^2 = n^2 + m^2 + m \cdot n$. All energies are then integer multiples of the fundamental energy.

The free-space evolution of the density in the traditional Talbot effect can be mapped to the evolution in a HO by considering the dynamics of the $\tilde{x}$ operator. In free space we have after a time of flight nT_{Talbot} corresponding to the nth Talbot revival:

$$\tilde{x}(nT_{\text{Talbot}}) = \tilde{x}_0 + \omega nT_{\text{Talbot}}\tilde{p}_0 \tag{S27}$$

and in the trap:

$$\tilde{x}(t_{\text{ho}}) = \cos(\omega t_{\text{ho}})\tilde{x}_0 + \sin(\omega t_{\text{ho}})\tilde{p}_0 \tag{S28}$$

It follows that for $t_{\text{ho}} = \frac{1}{\omega}\arctan(\omega nT_{\text{Talbot}})$

$$\tilde{x}(t_{\text{ho}}) = \frac{1}{M'}\tilde{x}(nT_{\text{Talbot}}) \tag{S29}$$

with $M' = \sqrt{1 + (\omega nT_{\text{Talbot}})^2} = 1/\cos(\omega t_{\text{ho}})$.
This shows that the dynamics in the trap can be mapped to the dynamics in free-space upon rescaling positions with a factor $1/M'$ and rescaling of the evolution times via the relation

$$t_{\text{ho}} = \frac{1}{\omega}\arctan(\omega nT_{\text{Talbot}}). \tag{S30}$$

The distribution in the trap at time t_{ho} corresponding to the nth Talbot revival can be then magnified by a factor M just by letting the system remain in the trap for an additional $\sim T/4$ and subsequent t_{tof} expansion. One gets in the end:

$$\tilde{x} = \frac{M}{M'}\tilde{x}(nT_{\text{Talbot}}) \sim \omega t_{\text{tof}}\tilde{x}(nT_{\text{Talbot}}) \tag{S31}$$

(since typically $\omega T_{\text{Talbot}} \ll 1$ and $\omega t_{\text{tof}} \gg 1$).

As derived in ref.[S24] for the 1D case, the strength of the Talbot revivals is a measure of the phase correlation function. We argue that the decay of the contrast with the order of the revival is related to the phase correlation function also in the 2D case. We note that the quantum gas magnifier would allow access the contrast and therefore the phase correlation function in a spatially resolved manner. This is particularly relevant for inhomogeneous systems, as typically the case for harmonically trapped quantum gases.

Imaging of coherent wavepackets away from the focusing condition of the quantum gas magnifier can be used to gain information on phase profiles of the wavefunction, where phase fluctuations of low-dimensional systems are transformed into density fluctuations[S25,S26]. As an example, we suggest it could be used to detect domains of magnetic order encoded in the condensate phases[S27].

These measurement would take place by adding an evolution time in the HO, followed by the magnification whose first step is also an evolution in the HO itself: we notice that one could also measure negative waiting times simply by a total wait time in the HO smaller than required from the precise imaging of the density $\omega t_{\text{ho}} < \arctan(\omega t_{\text{tof}})$. Measuring for both positive and negative times would allow detection of time reversal asymmetry, a strong hint for chiral states.

---

* klaus.sengstock@physnet.uni-hamburg.de

[S1] Murthy, P. A. *et al.* Matter-wave Fourier optics with a strongly interacting two-dimensional Fermi gas. *Phys. Rev. A* **90**, 043611 (2014).

[S2] Tung, S., Lamporesi, G., Lobser, D., Xia, L. & Cornell, E. A. Observation of the presuperfluid regime in a two-dimensional Bose gas. *Phys. Rev. Lett.* **105**, 230408 (2010).

[S3] Weinberg, M. *et al.* Symmetry-broken momentum distributions induced by matter-wave diffraction during time-of-flight expansion of ultracold atoms. *Phys. Rev. A* **93**, 033625 (2016).

[S4] Thomas, C. K., Barter, T. H., Leung, T.-H., Daiss, S. & Stamper-Kurn, D. M. Signatures of spatial inversion asymmetry of an optical lattice observed in matter-wave diffraction. *Phys. Rev. A* **93**, 063613 (2016).

[S5] Kovachy, T. *et al.* Matter wave lensing to picokelvin temperatures. *Phys. Rev. Lett.* **114**, 143004 (2015).

[S6] Hueck, K. *et al.* Two-dimensional homogeneous Fermi gases. *Phys. Rev. Lett.* **120**, 60402 (2018).

[S7] Chomaz, L., Corman, L., Yefsah, T., Desbuquois, R. & Dalibard, J. Absorption imaging of a quasi-two-dimensional gas: A multiple scattering analysis. *New J. Phys.* **14**, 055001 (2012).

[S8] Endres, M. *et al.* Observation of correlated particle-hole pairs and string order in low-dimensional Mott insulators. *Science* **334**, 200–203 (2011).

[S9] Bücker, R. *et al.* Single-particle-sensitive imaging of freely propagating ultracold atoms. *New J. Phys.* **11**, 103039 (2009).

[S10] Fuhrmanek, A. *et al.* Imaging a single atom in a time-of-flight experiment. *New J. Phys.* **12**, 053028 (2010).

[S11] Picken, C. J., Legaie, R. & Pritchard, J. D. Single atom imaging with an sCMOS camera. *Appl. Phys. Lett.* **111**, 164102 (2017).

[S12] Bergschneider, A. *et al.* Spin-resolved single-atom imaging of ^{6}Li in free space. *Phys. Rev. A* **97**, 063613 (2018).

[S13] Lawall, J. *et al.* Three-dimensional laser cooling of helium beyond the single-photon recoil limit. *Phys. Rev. Lett.* **75**, 4194–4197 (1995).

[S14] Cayla, H. *et al.* Single-atom-resolved probing of lattice gases in momentum space. *Phys. Rev. A* **97**, 061609 (2018).

[S15] Joffe, M. A., Ketterle, W., Martin, A. & Pritchard, D. E. Transverse cooling and deflection of an atomic beam inside a Zeeman slower. *J. Opt. Soc. Am. B* **10**, 2257–2262 (1993).

[S16] Preiss, P. M., Ma, R., Tai, M. E., Simon, J. & Greiner, M. Quantum gas microscopy with spin, atom-number, and multilayer readout. *Phys. Rev. A* **91**, 041602 (2015).

[S17] Boll, M. *et al.* Spin- and density-resolved microscopy of antiferromagnetic correlations in Fermi-Hubbard chains. *Science* **353**, 1257–1260 (2016).

[S18] Qu, A., Evrard, B., Dalibard, J. & Gerbier, F. Probing spin correlations in a Bose-Einstein condensate near the single-atom level. *Phys. Rev. Lett.* **125**, 033401 (2020).

[S19] Nelson, K. D., Li, X. & Weiss, D. S. Imaging single atoms in a three-dimensional array. *Nature Phys.* **3**, 556–560 (2007).

[S20] Elíasson, O. *et al.* Spatial tomography of individual atoms in a quantum gas microscope. *Phys. Rev. A* **102**, 053311 (2020).

[S21] Clauser, J. F. & Li, S. Talbot-vonLau atom interferometry with cold slow potassium. *Phys. Rev. A* **49**, 2213–2217 (1994).

[S22] Chapman, M. S. *et al.* Near-field imaging of atom diffraction gratings: The atomic Talbot effect. *Phys. Rev. A* **51**, R14–R17 (1995).

[S23] Nowak, S., Kurtsiefer, C., Pfau, T. & David, C. High-order Talbot fringes for atomic matter waves. *Optics Letters* **22**, 1430 (1997).

[S24] Santra, B. *et al.* Measuring finite-range phase coherence in an optical lattice using Talbot interferometry. *Nature Commun.* **8**, 15601 (2017).

[S25] Dettmer, S. *et al.* Observation of phase fluctuations in elongated Bose-Einstein condensates. *Phys. Rev. Lett.* **87**, 160406 (2001).

[S26] Choi, J.-y., Seo, S. W., Kwon, W. J. & Shin, Y.-i. Probing phase fluctuations in a 2d degenerate Bose gas by free expansion. *Phys. Rev. Lett.* **109**, 125301 (2012).

[S27] Struck, J. *et al.* Quantum simulation of frustrated classical magnetism in triangular optical lattices. *Science* **333**, 996–999 (2011).
